# A general and atom-efficient continuous-flow approach to prepare amines, amides and imines via reactive *N*-chloramines

**DOI:** 10.3762/bjoc.14.196

**Published:** 2018-08-24

**Authors:** Katherine E Jolley, Michael R Chapman, A John Blacker

**Affiliations:** 1 School of Chemistry, Institute of Process Research and Development, University of Leeds, Leeds, LS2 9JT, United Kingdom; 2 School of Chemical and Process Engineering, University of Leeds, Leeds, LS2 9JT, United Kingdom

**Keywords:** continuous flow, CSTR, *N*-chloramine, synthetic methods, telescoping

## Abstract

Chloramines are an important class of reagents, providing a convenient source of chlorine or electrophilic nitrogen. However, the instability of these compounds is a problem which makes their isolation and handling difficult. To overcome these hazards, a continuous-flow approach is reported which generates and immediately reacts *N*-chloramines directly, avoiding purification and isolation steps. 2-Chloramines were produced from the reaction of styrenes with *N*-alkyl-*N*-sulfonyl-*N*-chloramines, whilst *N*-alkyl or *N,N’*-dialkyl-*N*-chloramines reacted with anisaldehyde in the presence of *t*-BuO_2_H oxidant to afford amides. Primary and secondary imines were produced under continuous conditions from the reaction of *N*-chloramines with base, with one example subsequently reduced under asymmetric conditions to produce a chiral amine in 94% ee.

## Introduction

*N*-Chloramines are versatile reagents, however, their availability is restricted by their stability, so useful would be in situ methods to produce and use them [1–2]. The continuous-flow methodology is useful in this context, enabling control over reaction exotherms and improved measures for containment. To evaluate the use of *N*-chloramines in the laboratory requires multiphase flow methods, and until recently these have been limited by the availability of suitable equipment. Microreactors have been used for mixing biphases and employ either static mixers or shaped chambers and channels that repeatedly split and mix the liquids [3–5]. These rely on flow rates within the mixing zone that are sufficient to overcome phase separation [6]. Actively mixed, multistage and variable residence time (*t*_res_) continuous stirred tank reactors (CSTRs) allow much lower flow rates and therefore longer *t*_res_ for slow reactions [7–8]. The use of CSTRs to carry out sequential or multistep reactions has been exploited by Ley and others [9–11]. The strategy is useful, since it has the potential to eliminate time-consuming and costly product isolations. In these systems, the reactants and products are fluids which are contacted with solid-supported reagents that after some time require regeneration, which is not convenient within chemical manufacture.

Chloramine itself is unstable, though has been produced safely at large scale using continuous-flow methods; in fact, chloramine has been used as an intermediate in the manufacture of hydrazine using the Raschig process [12–13]. *N*-Alkyl-*N*-chloramines are equally unstable, yet have only been prepared in batch via reaction of a primary or secondary amine with Cl_2_ gas, *N*-chlorosuccinimide, chloramine-T or hypochlorite salts [14–15]. Whilst Cl_2_ gas is atom efficient it is difficult to handle, with associated toxicity, and the acid byproduct which leads to *N*-chloramine hydrolysis [16]. On the other hand, *N*-chlorosuccinimide or chloramine-T are commonly employed, being commercially available, stable and straightforward to handle, though both exhibit poor atom economy [17–21]. Sodium hypochlorite (NaOCl) solutions are less widely used, yet readily available, economic and provide an atom efficient reagent for *N*-chloramine formation [22–24].

**Figure 1:**
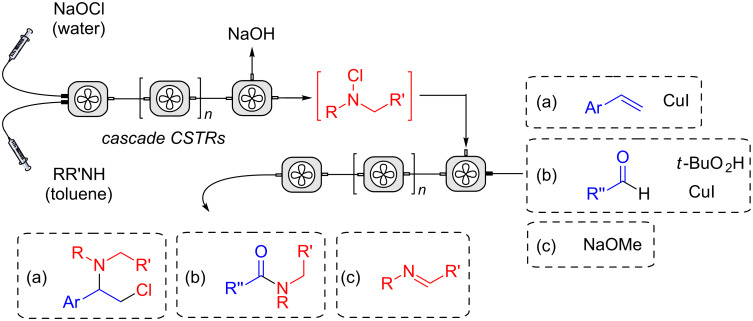
Continuous-flow process to produce and react *N*-chloramines.

A continuous-flow process for the oxidation of alcohols using NaOCl as a phase-transfer catalyst was recently reported [25]. We have published a communication that describes the continuous mixing of aqueous NaOCl and an organic solution of secondary amine, using either a tubular reactor with in-line static mixers or a single stage CSTR [26]. The reactor was selected to provide a *t*_res_ for optimal conversion. This was achieved according to reaction kinetics and hydrophobicity of the amine, which affects its partition between phases. Herein, we report improvements to this process and the use of *N*-alkyl-*N*-chloramine in subsequent continuous-flow reactions (Figure 1).

These reagents can be used as electrophilic or radical amination agents in a wide range of reactions [14]. In the present study, we opted to evaluate the addition of *N*-alkyl-*N*-chloramines with (a) alkenes to produce amines, (b) aldehydes to give amides, (c) reaction with a base to afford imines. Several alkenes are known to react with *N*-haloamines to form aziridines and other *N*-heterocycles. Typically, the reactions require a catalyst (e.g., Cu, I_2_) [17–18,27], whilst more active reagents such as chloramine-T with osmate catalysts have been used to make 1,2-aminoalcohols and diamines [28–33]. Improved methods for the formation of amides remain an important goal for the pharma industry. In this regard, the reaction of *N*-chloramine with aldehydes, *t*-BuO_2_H and iron or copper catalysts to give secondary and tertiary amides was reported in batch recently [33–34], though safety concerns upon scale-up makes this a useful reaction to translate to flow. Likewise, imines are an important class of compounds and are increasingly used as precursors to optically active amines [35–39]. Whilst normally prepared via a corresponding carbonyl compound, final dehydration can be problematic. The oxidation of a racemic amine and subsequent chiral reduction may offer a valuable alternative if coupled into a sequential flow protocol. There are reports on the formation of imines from *N*-chloramines using bases (e.g., NaOMe, KO*t*-Bu, NEt_3_ and NaOH) [40–45], with one specific study using this technique to racemise and resolve enantiopure tetrahydroquinolines [46–47], and another accessing an intermediate to the drug telaprevir [45]. Our study complements these findings, by supplying a continuous-flow oxidation–reduction sequence which telescopes both *N*-chloramine and imine intermediates to produce chiral amines.

## Results and Discussion

### *N*-Chloramine formation

**Figure 2:**
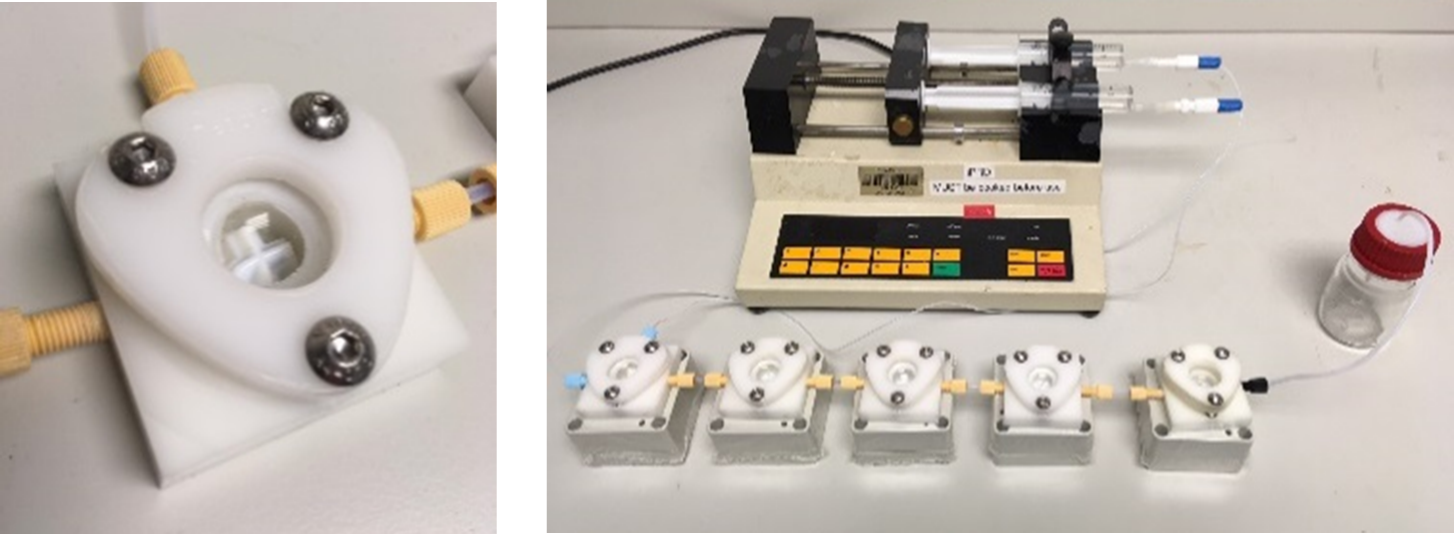
Left: Laboratory scale CSTR developed by our group [8]. Right: 5-stage CSTR configuration using co-feeds of amine in toluene and aqueous NaOCl.

*N*-(Di)alkyl-*N*-chloramines have been prepared in continuous organic–aqueous biphasic flow using either static mixers or a single-stage CSTR [26]. The choice of reactor and definition of *t*_res_ for this reaction is governed by both the thermodynamic phase partition parameter of reactants and mixing efficiency which control mass transfer between each phase (and thus, reaction rate). We decided to exploit a multi-stage cascade CSTR developed by our group recently [8], which enables efficient mixing over long *t*_res_ (Figure 2).

Using a 5-stage variant, various unsymmetrical *N*-chloramines were produced with unprecedented productivities (Table 1).

**Table 1:** Continuous *N*-chloramine formation.

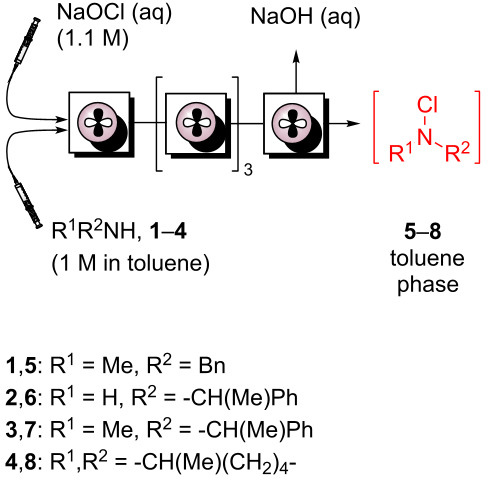						

entry	starting material	product	reactor^a^ (vol/mL)	*t*_res_ (min)	conversion (%)^b^	productivity (mol L^−1^ h^−1^)

1	**1**	**5**	SM (6)	20	89	1.3
2	**1**	**5**	CSTR^c^ (50)	25	100	1.2
3	**1**	**5**	CSTR^d^ (10)	5	94	5.6
4	**2**	**6**	CSTR^d^ (10)	10	92	2.8
5	**3**	**7**	CSTR^d^ (10)	30	93	0.9
6	**4**	**8**	CSTR^d^ (10)	10	100	3.0

^a^SM = static mixer. ^b^Determined by ^1^H NMR spectroscopy. ^c^1-Stage CSTR. ^d^5-Stage CSTR.

The rapid nature of this chlorination step makes in situ generation and consumption feasible in flow mode. Comparing Table 1, entries 1 and 3, the 5-stage CSTR, with one fifth the volume of that in Table 1, entry 2, provides a much shorter *t*_res_ than the in-line static mixer (SM) with comparable conversion of *N*-benzyl-*N*-methylamine (**1**) to the *N*-alkyl-*N*-chloramine **5** at steady state, representing a productivity value of 5.6 mol L^−1^ h^−1^. The same reactor geometry was used to chlorinate primary, secondary acyclic and cyclic amines **2**–**4** in conversions between 92–100%, with productivities ranging between 0.9–3 mol L^−1^ h^−1^ (Table 1, entries 4–6). In each case, separation of the product-rich toluene phase avoided *N*-chloramine isolation and allowed direct deployment in further reactions.

#### Reaction of *N*-chloramine with alkene

Initially our study tested the reaction of *N-*chloromorpholine (**16**) to styrene (**13**) varying Cu catalyst loading and a range of temperatures. The anti-Markovnikov addition product was observed with 10% CuI catalyst loading, at ambient temperature. However, it required 24 hours (see Supporting Information File 1, S1), and this slow reaction prevents sensible translation of the process into continuous flow. Despite trying alternative catalysts or other conditions no improvement was found. Instead, the more electron-poor *N*-chloro-*N*-methyl-*p-*toluenesulfonamide (**11**) was investigated as substrate. Differential scanning calorimetry (DSC) was used to assess the thermal stability of **11**, which melts at 78 °C and decomposes between 160–200 °C. This profile peaks at 188 °C, corresponding to an enthalpy of decomposition of −84.7 kJ mol^−1^ (see Supporting Information File 1, S2). A maximum safe operating temperature of 110 °C was implemented to avoid thermal decomposition and thermal runaway.

The direct reaction of *N*-chloramine **11**, or the benzyl-substituted variant **12**, led to a single regioisomer of the amine product in a lower reaction time than the analogous reaction using **16** (15 minutes vs 24 hours in batch mode; Supporting Information File 1, Table S1, entry 2 and Table 2, entries 1–5. The products **14** and **15**, prepared in batch, were isolated in 78 and 68% yield, respectively, and characterized (see Supporting Information File 1, S4). These standards enabled monitoring of the steady-state conversion in continuous flow by ^1^H NMR.

**Table 2:** Batch vs flow study of reaction of *N-*chloramine with styrene.

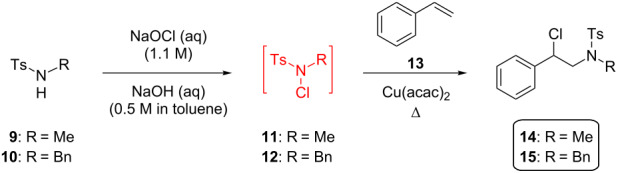						

entry	mode/substrate	catalyst (mol %)	temperature (°C)	time (min)	product	conversion (%)^a^

1	batch/**9**	2	100	15	**14**	62
2	batch/**9**	2	80	15	**14**	98
3	batch/**9**	2	rt	60	**14**	50
4	batch/**9**	0	100	60	**14**	100
5^b^	batch/**9**	0	100	60	**14**	0
6	batch/**10**	2	110	60	**15**	100
7^c^	CSTR/**9**	0	100	30 60 75	**14**	12 73 76
8^c^	CSTR/**10**	0	100	30 60 120	**15**	67 77 77

^a^Conversion measured by ^1^H NMR spectroscopy. ^b^Reaction carried out in either air or presence of TEMPO (1 equiv) led to the same result. ^c^1-Stage CSTR, co-feed with styrene in toluene and substrate in toluene/diglyme 3:1.

Following an optimization study, it was found that the Cu catalyst could be omitted when operating at 100 °C for 1 hour reaction time in batch, providing quantitative conversion to product (Table 2, entry 4). Conducting the same reaction under an atmosphere of air or in the presence of TEMPO, suppressed all product formation (Table 2, entry 5). Due to the safety concerns of scaling-up such a batch reaction, a heated single-stage CSTR was evaluated to immediately quench the *N*-chloramine. Flowing an aqueous solution of in situ generated **11** or **12** into a stream of toluene containing styrene (**13**) enabled the continuous production of alkylated amine products **14** and **15** (Table 2, entries 7 and 8, respectively). In each case the *t*_res_ was comparable with batch (reaction of **11** = 75 minutes, **12** = 60 minutes), with steady-state conversions or 76 and 77% observed, respectively.

#### Reaction of *N*-chloramine with aldehyde

Reaction of *N*-chloramines with aromatic and aliphatic aldehydes to form amides has been reported by Porcheddu [34]. Under these literature conditions, FeCl_3_ catalyst (0.15 mol %), *t*-BuO_2_H oxidant (3.6 equiv) and excess aldehyde **17** (5 equiv) were employed to react with dilute *N*-chloramine **16** (0.064 M in MeCN), delivering amide **18** in 77% conversion and 54% isolated yield. Our interests were to improve the productivity of this system, by exploiting higher concentrations of *N*-chloramine produced in flow mode (200 mM). Table 3 summarizes a comparative study between batch and continuous flow for this reaction.

**Table 3:** Batch vs flow study of reaction of *N*-chloramine with an aldehyde.

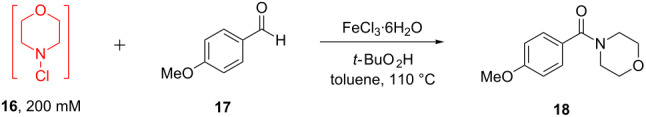					

entry	mode	FeCl_3_ (mol %)	equiv **17**/*t*-BuO_2_H	time (min)	conversion/yield (%)^a^

1^b^	batch	0.15	5/3.6	300	77/54
2^c^	batch	0.15	5/3.6	300	60
3	batch	15	5/3.6	60	100
4	batch	0	5/3.6	120	90
5	batch	15	5/0	120	10
6	batch	0	1/3.6	120	30
7	CSTR	0	5/5	100	70^d^
8	CSTR	5	5/5	100	96^d^

^a^Conversion measured by gas chromatography as the average of three runs. ^b^Literature conditions quoted as 88% [34]. ^c^[**16**] = 200 mM. ^d^Conversion recorded at steady state.

Initial tests involving 200 mM substrate concentration afforded amide **18** in 60% conversion (Table 3, entry 2). Increasing the catalyst loading to 15 mol % led to a quantitative conversion of **18** within 1 hour reaction time. Unexpectedly, a control reaction omitting the FeCl_3_ catalyst resulted in 90% conversion following a two-hour reaction time (Table 3, entry 4). Removing the *t*-BuO_2_H oxidant reduced the reaction rate significantly, leading to 10% conversion under otherwise identical conditions (Table 3, entry 5), whilst fewer equivalents of aldehyde **17** led to 30% product formation (Table 3, entry 6). Notably, other oxidants such as H_2_O_2_ and NaOCl failed to produce any amide product. Likewise, attempts to couple morpholine in place of its *N*-chloro derivative reached only 19% conversion.

**Figure 3:**
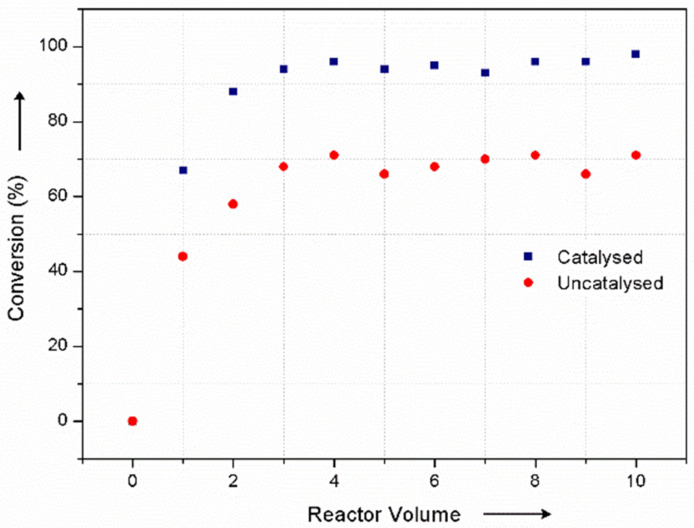
Continuous-flow amide **18** formation using 1-stage CSTR. Blue squares: FeCl_3_ included; red circles: FeCl_3_ not included.

Following the investigation of the batch reaction, it was transferred to a CSTR. Feeding 200 mM *N*-chloramine to meet a separate solution of aldehyde **17** (5 equiv) and *t*-BuO_2_H (5 equiv), a *t*_res_ of 100 minutes afforded amide **18** in 70% conversion at steady state. Under analogous conditions, FeCl_3_ (5 mol %) was included in the oxidant stream to give 96% steady-state conversion to **18** (Figure 3). This data represents productivities of 19 and 26 g L^−1^ h^−1^ for the uncatalysed and FeCl_3_-catalyzed amide formation, respectively (Supporting Information File 1, S4).

#### Reaction of *N*-chloramine with base

The base-induced dehydrochlorination of *N*-chloramines is a facile route to imines, which may be used for further functionalization. Our study began by examining a host of bases to convert *N*-chloramine **5** to benzylidene(methyl)amine (**19**) as a model reaction system (Table 4).

**Table 4:** Batch optimization study of the dehydrochlorination of *N*-chloramines.

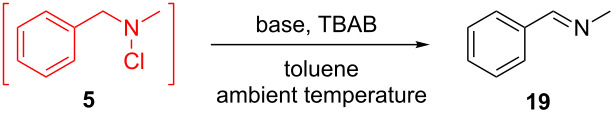					

entry	base	catalyst	solvent	time (h)	conversion (%)^a^

1	NEt_3_ (5 equiv)	none	toluene	42	92
2	KO*t*-Bu (5 equiv)	none	MeOH	15	90
3	NaOMe (2 equiv)	none	toluene/MeOH 1:1	2	100
4	NaOMe (10 equiv)	none	toluene/MeOH 1:1	1	100
5	NaOMe (1 equiv)	none	toluene/MeOH 1:1	1	47
6	NaOMe (5 equiv)	none	toluene	1	100
7	NaOH 25% aq	TBAB^b^	toluene/water 1:1	1	83
8	NaOH 25% aq	TBAB^b^	toluene/water 1:1	3	100
9	NaOH 40% aq	TBAB^b^	toluene/water 1:1	1	50
10	NaOH 25% aq	none	toluene/MeOH (1%)	19	0
11	NaOH 25% aq	none	toluene/MeOH (20%)	19	0

^a^Measured by ^1^H NMR spectroscopy. ^b^Reaction temperature = 60 °C.

To achieve a complete conversion, NEt_3_ was required in large excess (5 equiv) over 42 hours, which proved unsuitable for continuous flow (Table 4, entry 1). Whilst KO*t*-Bu and NaOMe bases allowed rapid imine formation (Table 4, entries 2–6), though their low solubility in MeOH or toluene would require slurry pumping in flow mode which is undesirable. In addition, the isolation procedure is not straightforward, requiring multiple unit operations. To avoid this, a phase-transfer catalyst (TBAB) was used along with NaOH (Table 4, entries 7–9). This reagent, in a toluene/water mixture, promoted full conversion to imine **19** (Table 4, entry 8). The separation of the toluene phase provided the imine product, which may be deployed directly in further reactions.

To validate the batch protocol *N*-chloramines **5** and **7** underwent smooth dehydrochlorination to produce imines **19** and **20** in 83 and 100% conversion after 1 hour (Table 5, entries 1 and 2). The cyclic *N*-chloramine **8** was converted in batch mode to the corresponding imine **21**, though required 18 hours to reach 84% conversion (Table 5, entry 3). The rapid nature of the imine formation prompted us to investigate a fully continuous protocol to both *N*-chlorinate and subsequently dehydrochlorinate amines, which would represent a mild and atom-efficient alternative method of amine oxidation. A 5-stage cascade CSTR was employed to link *N*-chloramine generation with base-promoted imine formation. A 1 M stream of *N*-chloramine **5** in toluene was mixed in the first CSTR with separate flows of aqueous NaOH and TBAB (10 mol % relative to substrate) and *t*_res_ was adjusted by changing the number of subsequent CSTR chambers (*n*) (Table 5, entry 1). It is noteworthy that attempts to mix NaOH and TBAB solutions via a T-piece prior to the mixing chamber were not successful, as a precipitate forms from the mixture leading to reactor blockage. A quantitative conversion of **5** to imine **19** was realized using the NaOH/TBAB protocol with a *t*_res_ of 2 hours with good productivity (0.25 mol L^−1^ h^−1^, Table 5, entry 1). Under analogous conditions, *N*-chloramine **7** was converted to imine **20** in 88% conversion, which could be improved to 99% conversion by extending *t*_res_ to 3 hours (Table 5, entry 2). However, the same conditions proved only able to convert 19% of the *N*-chloramine **8** at steady state with *t*_res_ of 2 h (Table 5, entry 3). To achieve a higher conversion an impractical *t*_res_ would be required if the same batch conditions were used. In this regard, the use of heated CSTRs would be useful to explore.

**Table 5:** Batch vs flow study of the dehydrochlorination step.

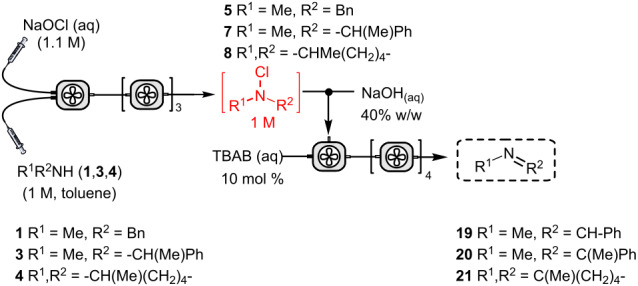				

entry	product	mode	*t*_res_ (h)	conversion (%)^a^

1	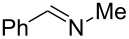 **19**	batch flow	1 2	83 100
2	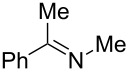 **20**	batch flow flow	1 2 3	100 88 99
3	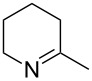 **21**	batch flow	18 2	84 19

^a^Measured by ^1^H NMR spectroscopy.

**Scheme 1:**
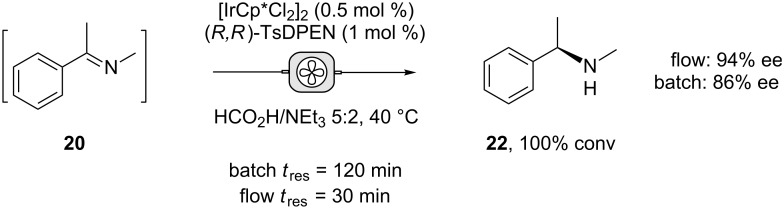
Continuous-flow transfer hydrogenation of in situ generated imines.

The formation of both imines **20** and **21** are of interest as an asymmetric reduction would give an optically pure amine. To demonstrate this, imine **20**, formed in situ, underwent asymmetric-transfer hydrogenation in both batch and flow modes, using [IrCp*Cl_2_]_2_ as catalyst with the ligand (*R,R*)-TsDPEN, using the hydrogen-donor reagent formic acid/triethylamine (Scheme 1).

Under batch conditions, a *t*_res_ of 120 minutes gave quantitative reduction of the imine, affording the *R*-isomer in 86% ee*.* Translating the procedure to continuous flow, a fresh solution of imine **20** and catalyst mixture were pumped into a heated CSTR over a 30-minutes *t*_res_, affording chiral amine **22** in 94% ee with complete conversion. It is unclear why a higher optical activity was seen using continuous flow. However, it is known that [IrCp*Cl_2_]_2_ can slowly racemise this amine which may be more of a problem in batch with the longer reaction time [48].

## Conclusion

A continuous-flow approach to prepare and handle unstable *N*-chloramines is reported. The method exploits the superior mixing of a CSTR compared with classical batch, to enable fast *N*-chlorination of amines under biphasic conditions. By virtue of a flowing solution, the in situ generated chloramines may be transferred directly into new reaction media, with examples of (i) addition to an alkene to form a new C–N and C–Cl bond, (ii) reaction with aldehyde to produce amides, and (iii) dehydrochlorination with a base to afford imines reported within our study. Of these examples, the latter was further explored by immediate asymmetric-transfer hydrogenation of an in situ formed imine under continuous-flow conditions, as a potentially productive route to chiral amines.

## Supporting Information

File 1 Details of reactor assembly, NaOCl titration and NMR spectra.
