# Supplementary material for: A general and atom-efficient continuous-flow approach to prepare amines, amides and imines via reactive N-chloramines
Source: Beilstein J Org Chem. 2018 Aug 24;14:2220–8. doi: 10.3762/bjoc.14.196 (PMC6122332; doi:10.3762/bjoc.14.196)
Supplement: File 1 — Details of reactor assembly, NaOCl titration and NMR spectra. [file Beilstein_J_Org_Chem-14-2220-s001.pdf]

Supporting Information

for

A general and atom-efficient continuous-flow approach to prepare amines, amides and imines via reactive *N*-chloramines

Katherine E. Jolley,[1] Michael R. Chapman,[1] and A. John Blacker*[1,2]

Address: [1]School of Chemistry, Institute of Process Research and Development, University of Leeds, Leeds, LS2 9JT, United Kingdom and [2]School of Chemical and Process Engineering, University of Leeds, Leeds, LS2 9JT, United Kingdom

Email: A. John Blacker - j.blacker@leeds.ac.uk

*Corresponding author

Details of reactor assembly, NaOCl titration and NMR spectra

Table of contents

S1. Batch reaction screening for addition of *N*-chloromorpholine to styrene S2
S2. Differential scanning calorimetry (DSC) of *N*-chloro-*N*-methyl-*p*-toluenesulfonamide S3
S3. Kinetic study of *N*-(4-methoxybenzoyl)morpholine formation S4
S4. Experimental Section S5
    S4.1 General procedures S5
    S4.2 Single-stage heated CSTR assembly S14
    S4.3 Multi-stage CSTR assembly S15
    S4.4 NaOCl titration S16
S5. GC conditions and calibration profiles S16
S6. Relevant ^{1}H and ^{13}C NMR spectra S19
S7. References S31

S1. Batch reaction screening for addition of *N*-chloromorpholine to styrene

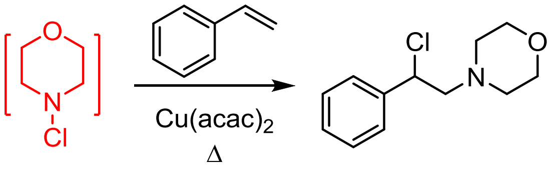

Table S1. Batch addition of N-chloromorpholine to styrene. Reaction time 24 h

	Eq. N-Chloromorpholine	Catalyst	Temp (°C)	Conversion (%)[a]
1	1	None	RT	0
2	1	CuI, 10 mol%	RT	60
3	1.5	CuI, 10 mol%	RT	43
4	1	CuI, 5 mol%	RT	60
5	1	$Cu(OTf)_2$ 10 mol%	RT	40
6	1	$CuBr_2$	RT	0
7	1	$FeCl_3.6H_2O$	RT	0
8	None, 1 eq. morpholine	CuI, 10 mol%	RT	0
9[b]	1	CuI, 10 mol%	RT	30
10[c]	1	CuI, 10 mol%	RT	79
11[d]	1	CuI, 10 mol%	RT	12
12[d]	1	CuI, 10 mol%	60	13
13[d]	1	CuI, 10 mol%	110	14
14[e]	2	CuI, 10 mol%	60	0

[a] Conversion determined by ^{1}H NMR spectroscopy. [b] 1:1 toluene:water as solvent. [c] Additional CuI 10 mol% and 1 eq. N-chloromorpholine added after 8 hours. [d] Reaction time = 1 hour. [e] Reaction time = 4 hours.

Various other non-precious metal catalysts reported in the literature were examined ($Cu(OTf)_2$[1], $FeCl_3$[2]), however, CuI proved most active despite long reaction times and poor conversion. Increasing temperature (entries 11–14) or concentration of *N*-chloromorpholine (entries 3 and 14) did not increase overall conversion. Substitution of *N*-chloromorpholine with morpholine did not afford any trace of product. The addition of water to the reaction solvent impeded conversion, preventing a one-pot procedure. Interestingly however, addition of further *N*-chloromorpholine and CuI after 8 hours reaction time led to increased conversion (entry 10, 79%), suggesting catalyst and/or substrate degradation may be taking place.

S2. Differential scanning calorimetry (DSC) of *N*-chloro-*N*-methyl-*p*-toluenesulfonamide

N-Chloro-*N*-methyl-*p*-toluenesulfonamide (2.3 mg, 0.01 mmol) was analysed by DSC. The sample was heated from 10 → 250°C at a step-rate of 10 °C min^{-1}.

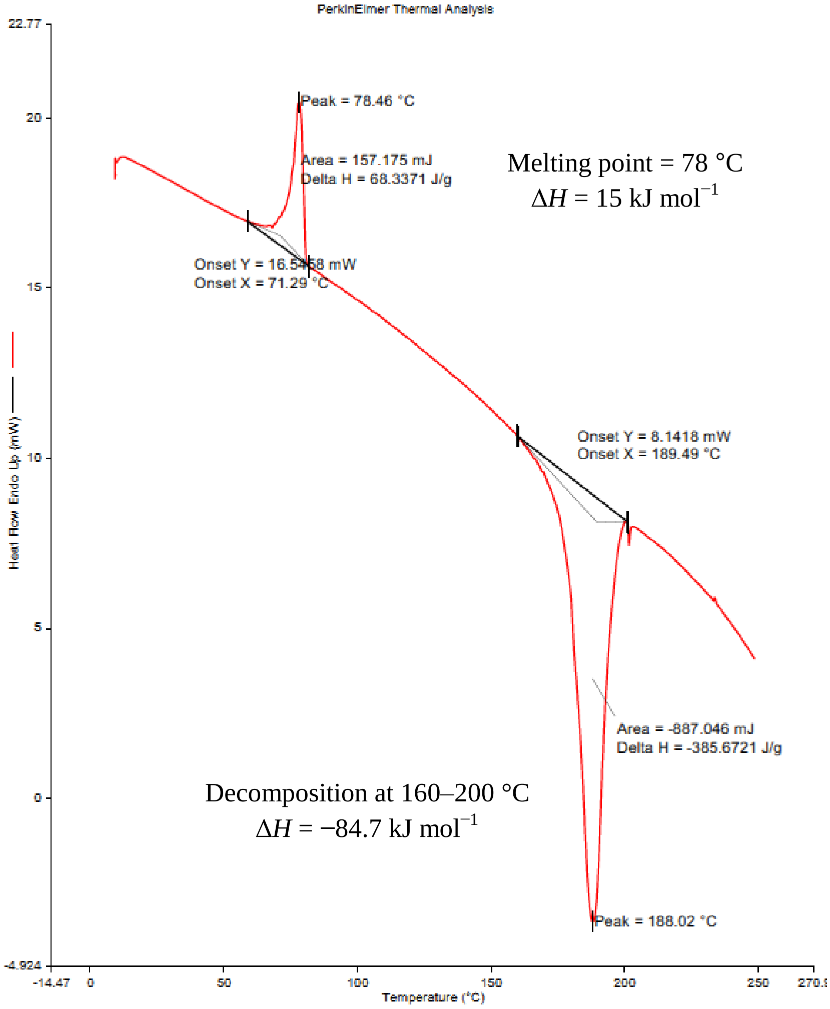

Figure S1. DSC curve for *N*-chloro-*N*-methyl-*p*-toluenesulfonamide.

S3. Kinetic study of *N*-(4-methoxybenzoyl)morpholine formation

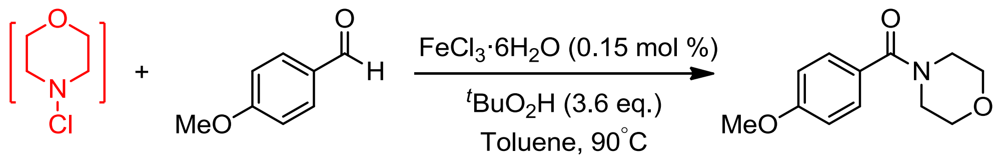

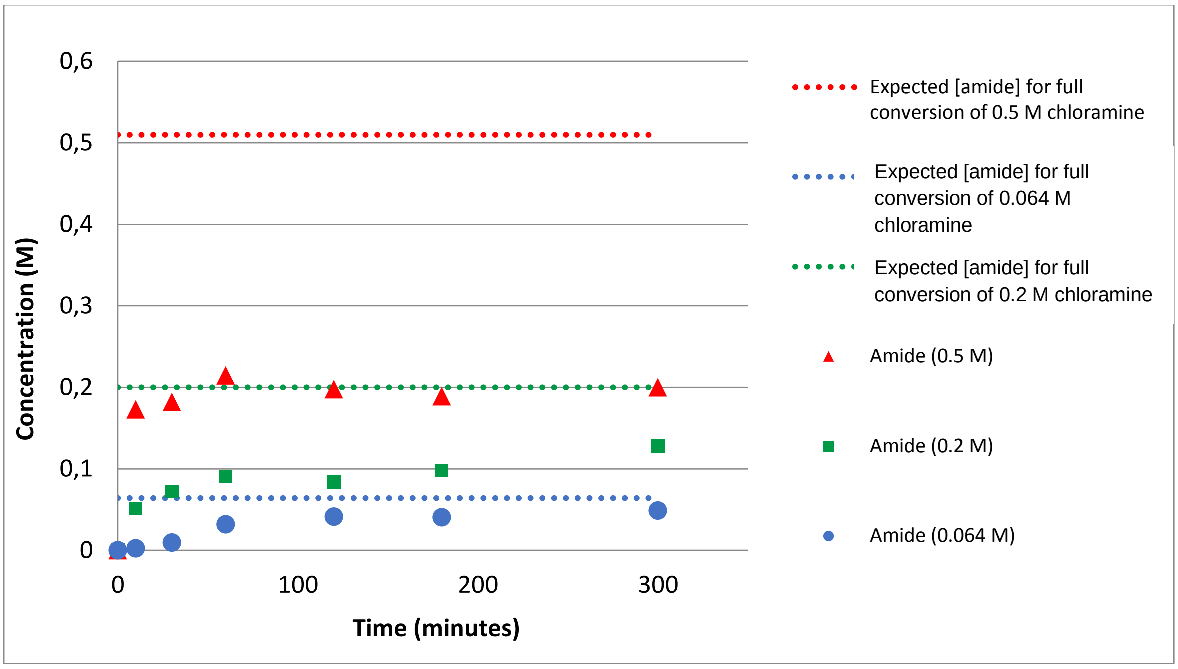

Figure S2. Effect of reaction concentration on formation of *N*-(4-methoxybenzoyl)morpholine.

S4. Experimental Section

S4.1 General procedures: All commercially obtained chemicals were used as received from suppliers without purification. Sodium hypochlorite was obtained as an aqueous solution with 10–15% available chlorine purchased from Sigma-Aldrich. The accurate NaOCl concentration was determined by titration (see below) and diluted to the required concentration with DI water. $CDCl_3$ purchased from Sigma-Aldrich was used for NMR analysis. NMR spectra (^{1}H and ^{13}C) were obtained on either a Bruker Advance 500 MHz or 400 MHz spectrometer and were referenced to either TMS or $CHCl_3$. GC analysis was carried out on an Agilent HP 6890 with FID. Differential scanning calorimetry experiments were carried out using a Perkin Elmer Pyris 1 differential scanning calorimeter. Mass spectrometry analysis was carried out using a Bruker HCT ion trap mass spectrometer coupled with an Agilent 1100 HPLC (LRMS) or a Bruker MaXis Impact spectrometer with Ultimate 3000 UPLC (HRMS). IR spectroscopy was carried out using a Bruker ALPHA FT-IR spectrometer. Melting points were determined using a Stuart Melting Point Aparatus SMP30. For continuous reactions, Harvard syringe pumps (model 981074) or Watson Marlow 323S peristaltic pumps were used.

General procedure for continuous *N*-chloramine synthesis using the single-stage CSTR: Previously reported in reference [3]. The required number of reactors were connected in series using PTFE tubing ($^1/_8$" outer diameter, $^1/_{16}$" inner diameter). A 1 M solution of the amine in toluene was prepared along with a 1.1 M aqueous solution of NaOCl. The solutions were pumped into the stirred reactor at equal flow rates to give the required residence time. The reaction solution was collected from the reactor in reactor volume fractions. The organic phase of each fraction was separated and analysed directly by ^{1}H NMR spectroscopy as a solution in toluene (100 μL organic solution added to an NMR tube containing 4.5 cm^3 $CDCl_3$). Due to the hazards associated with *N*-chloramines and their volatility, isolation of the product is not advised. Products were therefore obtained as solutions in toluene. Due to the presence of toluene, IR spectra could not be obtained for the compounds.

N-Chloro-*N*-methylbenzylamine (**5**): Data compares with that previously reported for this compound [3, 5]. ^{1}H NMR ($CDCl_3$, 300 MHz) δ ppm 7.32-7.28 (m, 5H, CHAr), 3.99 (s, 2H, CH_2), 2.88 (s, 3H, CH_3); ^{13}C NMR ($CDCl_3$, 125 ppm) δ ppm 139.37 (CAr), 128.58 (2 × CHAr), 128.49 (2 × CHAr), 127.32 (CHAr), 55.85 (CH_2), 35.68 (CH_3). The compound was not stable during mass spectrometry analysis and hence LCMS and HRMS data were not obtainable.

N-Chloro-α-methylbenzylamine (**6**): Data compares with that previously reported for this compound [3, 5]. ^{1}H NMR ($CDCl_3$, 500 MHz) δ ppm 7.55-7.49 (m, 5H, CHAr), 4.44-4.43 (br m, 1H, NH), 4.32-4.28 (m, 1H, CH), 1.66 (d J = 7.5 Hz, 3H, CH_3); ^{13}C NMR ($CDCl_3$, 125 ppm) δ ppm 142.15 (CAr), 128.77 (2 × CHAr), 128.23 (CHAr), 127.08 (2 × CHAr), 64.75 (CH), 21.32 (CH_3). The compound was not stable during mass spectrometry analysis and hence LCMS and HRMS data were not obtainable.

N-Chloro-*N*,α-dimethylbenzylamine (**7**): Data compares with that previously reported for this compound [3, 6]. ^{1}H NMR ($CDCl_3$, 500 MHz) δ ppm 7.34-7.29 (m, 5H, CHAr), 3.89 (q J = 6.6 Hz, CH), 2.80 (s, 3H, CH_3NCl), 1.54 (d J = 6.6 Hz, CH_3CH) ; ^{13}C NMR ($CDCl_3$, 125 ppm) δ ppm 141.88 (CAr), 128.42 (2 × CHAr), 127.85 (CHAr), 127.67 (2 × CHAr), 71.68 (CH), 49.90 (CH_3), 20.64 (CH_3). The compound was not stable during mass spectrometry analysis and hence LCMS and HRMS data were not obtainable

N-Chloro-2-methylpiperidine (**8**): Data compares with that previously reported for this compound, however due to volatility and stability the product could not be separated entirely from its toluene solution [3,5,7]. ^{1}H NMR ($CDCl_3$, 500 MHz) δ ppm 3.67-3.65 (m, 1H, CH_2NCl), 3.09-3.04 (m, 1H, CH^AH^B), 2.82 (br s, 1H, CH^AH^B), 2.82 (br s, 1H, CH), 1.84-1.61 (m, 4H, 2 × CH_2), 1.57-1.44 (m, 2H, CH_2), 1.38 (d J = 6.1 Hz, CH_3); ^{13}C NMR ($CDCl_3$, 125 ppm) δ ppm 66.67 (CH_2), 64.39 (CH_2), 35.79 (CH_2), 27.89 (CH_2), 24.01 (CH_3). The compound was not stable during mass spectrometry analysis and hence LCMS and HRMS data were not obtainable.

Batch formation of *N*-benzyl-*p*-toluenesulfonamide (**10**): (i) To benzylamine (8.0 g, 74.6 mmol), cooled to 0 °C, was added *p*-toluenensulfonyl chloride (14.3 g, 74.6 mmol) and triethylamine (11.3 g,

112 mmol) in DCM (72 mL). The reaction mixture was stirred and allowed to warm to room temperature until complete conversion was observed by TLC (silica gel, 1:1 EtOAc/petroleum ether 40–60). Then, saturated aqueous $NaHCO_3$ was added to the reaction solution and the product was extracted with DCM (3 × 50 mL). The DCM phases were combined, dried over Na_2SO_4, filtered and the solvent removed by rotary evaporation to leave the product as a white solid (17.9 g, 68.5 mmol, 92%). ^{1}H NMR ($CDCl_3$, 500 MHz) δ_H (300 MHz, $CDCl_3$) 7.75 (2H, d J = 8.3 Hz, CHAr), 7.31-7.24 (5H, m, CHAr), 7.20-7.18 (2H, m, CHAr), 4.89 (1H, t J = 6.1 Hz, NH), 4.10 (2H, d J = 6.2 Hz, CH_2), 2.43 (3H, s, CH_3).

N-Chloro-*N*-methyl-*p*-toluenesulfonamide (**11**). Prepared according to the general procedure in reference [3]. 1) Tube reactor with 4 static mixers, a 6 mL reactor volume and a residence time of 3 min. 1.5 M NaOCl aq. at 1 mL/min and 1 M *N*-benzylmethylamine in EtOAc at 1 mL/min. 2) CSTR with a reactor volume of 50 mL, and residence time of 25 min. 1.1 M NaOCl aq. at 1 mL/min and 1 M *N*-methyl-*p*-toluenesulfonamide in toluene at 1 mL/min were used. The product was isolated for each reactor volume by separation of the organic phase for each reactor volume of solution and removal of the solvent by rotary evaporation to give a white solid (1 reactor volume gives 666 mg, 3.0 mmol, quantitative yield). ^{1}H NMR (CDCl3, 500 MHz) δ ppm 7.82 (d, J=8.4 Hz, 2H, CHAr), 7.42 (d, J=8.4 Hz, 2H, CHAr), 3.09 (s, 3H, NCH3), 2.48 (s, 3H, Ar-CH3); 13C NMR (CDCl3, 125 MHz) δ ppm 145.75 (CAr), 129.80 (2CHAr), 129.78 (2 CHAr), 128.28 (CAr), 45.51 (NCH3), 21.70 (ArCH3).

N-Benzyl-*N*-chloro-*p*-toluenesulfonamide (**12**). To *N*-benzyl-*p*-toluenesulfonamide (2.1 g, 8.0 mmol) was added toluene (8 mL) and the solution cooled to 0 °C. Aqueous NaOCl (0.8 M, 20 mL, 16 mmol) was slowly added. The reaction mixture was stirred until complete consumption of starting material by TLC (silica gel, 1:1 EtOAc/petroleum ether 40–60) and ^{1}H NMR spectroscopy. The organic phase of the reaction solution was separated. The aqueous phase was washed with EtOAc (10 mL). The organic phases were combined, dried over Na_2SO_4, filtered and the solvent removed by rotary evaporation to leave the pure product as a white solid (1.6 g, 5.4 mmol, 68%). Mp 131-132°C; IR ν_{max} 3267.42, 3066.4, 3030.9, 2967.7, 2919.9, 1594.2, 1166.2, 1084.2, 1010.8, 846.0, 799.5, 787.1, 758.9, 701.6, 625.5, 537.0 cm^{-1}; ^{1}H NMR ($CDCl_3$, 500 MHz) δ ppm 7.89 (d, J = 8.3 Hz, 2H, CHAr), 7.43

(2H, d J = 8.2 Hz, CHAr), 7.35-7.34 (5H, m, CHAr), 4.35 (2H, s, CH_2), 2.50 (3H, s, CH_3); ^{13}C NMR ($CDCl_3$, 125 MHz) δ ppm 145.63 (CAr), 133.73 (CAr), 129.87 (2 × CHAr), 129.67 (2 × CHAr), 129.09 (2 × CHAr), 128.63 (2 × CHAr), 60.53 (CH_2), 21.78 (CH_3). Compound is present as amine by mass spectrometry: LRMS: 284.6 (M^+ - Cl + H + Na) HRMS m/z (ESI): M^+ - Cl + H + Na, 284.0701 $C_{14}H_{15}NNaO_2S$ requires M 284.0721.

General procedure for batch reaction of *N*-chloramine with styrene (**13**): The desired quantity of a 1 M solution of *N*-chloromorpholine in toluene was purged with nitrogen and to it was added styrene (required equivalents) and the catalyst and ligand as required (1–10 mol %). The reaction mixture was stirred under nitrogen at room temperature for 24 hours. Following this, toluene was removed by rotary evaporation. The residue was purified by column chromatography to give the product.

General procedure for continuous reaction of *N*-chloramine with styrene (**13**). A round-bottomed flask connected to a reflux condenser and nitrogen supply. A 2 M solution of styrene in toluene containing $Cu(acac)_2$ (2 mol %) and 1,10-phenanthroline (2 mol %) was prepared and fed into the reactor by pump 1. A second solution of *N*-chloramine (2 M in toluene/diglyme 3:1) was fed by pump 2. Pump 3 was used to pump the reaction solution out of the reactor. The flow rates of the three pumps were chosen to give the required reactor volume and residence time for the reaction, and so that the flow rate of pump 3 (outflow from reactor) equalled the total flow rate of pumps 1 and 2 (reactor input). The reactor was heated to 100 °C and stirred under nitrogen. The reaction solution was collected in reactor volume (residence time) fractions. A small sample from each fraction was removed and analysed by ^{1}H NMR spectroscopy to monitor conversion. Fractions collected at steady state were combined and the product isolated as described for the general batch procedure above.

4-(2-Chloro-2-phenylethyl)-morpholine: Purification by column chromatography (silica gel, 0-100% EtOAc in petroleum ether 40-60) to give the product as an orange oil. For a reaction with 41% conversion by ^{1}H NMR spectroscopy, isolation by chromatography gave the pure product in 15% yield. IR ν_{max} 2958.7, 2854.7, 2809.2, 1494.0, 1453.0, 1307.8, 1146.5, 1113.6, 1006.4, 866.4, 763.1, 696.8, 530.3 cm^{-1}; ^{1}H NMR (500 MHz, $CDCl_3$) δ ppm 7.40-7.26 (5H, m, CHAr), 4.97 (1H, d J = 7.7 Hz, CH), 3.69-3.66 (4H, m, 2 × CH_2N), 3.02 (1H, dd J = 13.5, 8.0 Hz, $CHCH^AH^BN$), 2.83 (1 H, dd J =

13.5, 6.1 Hz, $CHCH^AH^BN$), 2.53-2.48 (4H, m, 2 × CH_2O); ^{13}C NMR (125 MHz, $CDCl_3$) δ ppm 140.34 (CAr), 128.61 (2 × CHAr), 128.45 (CHAr), 127.29 (2 × CHAr), 66.85 (2 × CH_2N), 66.48 (CH), 59.95 (CH_2), 53.72 (2 × CH_2O); LRMS m/z (APCI+) 226.1 (M^+ + H), 190.1 (M^+ – Cl – H); HRMS m/z (ESI): M^+ + H, 226.9505 $C_{12}H_{17}ClNO$ requires M 226.0999.

N-(2-Chloro-2-phenylethyl)-*N*,4-dimethylbenzenesulfonamide (**14**): Purification by column chromatography (silica gel, 0-60% EtOAc in petroluem ether 40-60) to give the product as a white solid. For a reaction with 93% conversion by ^1H NMR spectroscopy, isolation by chromatography gave the pure product in 77% yield. Mp 56-57.5°C; IR ν_{max} 3063.6, 3030.7, 2918.6, 1596.9, 1453.9, 1330.8, 1156.5, 1087.5, 990.4, 934.0, 743.5, 713.0, 694.0, 646.8, 548.0 cm^{-1}; ^1H NMR (500 MHz, $CDCl_3$) δ ppm 7.64 (2H, d J = 8.3 Hz, $CHArCSO_2$), 7.43-7.29 (7H, m, CHAr), 5.11 (1H, t J = 7.3 Hz, CHCl), 3.59 (1H, dd J = 14.5, 7.4 Hz, CH^AH^B), 3.41 (1H, dd J = 14.5, 7.3 Hz, CH^AH^B), 2.63 (3H, s, CH_3N), 2.42 (3H, s, CH_3Ar); ^{13}C NMR (125 MHz, $CDCl_3$) δ ppm 143.64 (CAr), 138.73 (CAr), 134.79 (CAr), 129.80 (2 × CHAr), 128.81 (2 × CHAr), 127.51 (2 × CHAr), 127.34 (2 × CHAr), 61.34 (CH), 58.05 (CH_2), 36.98 (CH_3), 21.53 (CH_3); LRMS m/z (ESI) 346.2 (M^+ + Na); HRMS m/z (ESI): M^+ + H, 324.0805 $C_{16}H_{19}ClNO_2S$ requires M 324.0825, M^+ + Na, 346.0625 $C_{16}H_{18}ClNNaO_2S$ requires M 346.0644.

N-(2-Chloro-2-phenylethyl)-*N*-benzyl-4-methylbenzenesulfonamide (**15**): Purification by column chromatography (silica gel, 0-75% EtOAc in petroleum ether 40-60). Mp 92-93°C; IR ν_{max} 3064.7, 3026.3, 1597.7, 1494.5, 1454.2, 1322.9, 1149.9, 951.8, 898.1, 812.5, 731.6, 696.6, 653.3 cm^{-1}; ^1H NMR (500 MHz, $CDCl_3$) δ ppm 7.70 (2H, d J = 8.3 Hz, CHAr), 7.32-7.28 (5H, m, CHAr), 7.25-7.23 (3H, m, CHAr), 7.19-7.17 (2H, m, CHAr), 4.98 (1H, t J = 7.3 Hz, CHCl), 4.4 (1H, d J = 15.2 Hz, CH^AH^BAr), 3.96 (1H, d J = 15.2 Hz, CH^AH^BAr), 3.69 (1H, dd J = 15.0, 7.2 Hz, $CHCH^AH^BN$), 3.53 (1H, dd J = 15.0, 7.5 Hz, $CHCH^AH^BN$), 2.44 (3H, s, CH_3); ^{13}C NMR (125 MHz, $CDCl_3$) δ ppm 143.66 (CAr), 138.66 (CAr), 136.92 (CAr), 135.36 (CAr), 129.80 (2 × CHAr), 128.75 (CHAr), 128.67 (2 × CHAr), 128.64 (2 × CHAr), 126.46 (2 × CHAr), 127.93 (CHAr), 127.54 (2 × CHAr), 127.39 (2 × CHAr), 60.66 (CHCl), 54.57 (CH_2Ar), 52.76 ($CHCH_2$), 21.57 (CH_3); LRMS m/z (ESI)

422.2 (M^+ + Na); HRMS m/z (ESI): M^+ + H, 400.1113 $C_{22}H_{23}ClNO_2S$ requires M 400.1138, M^+ + Na, 422.0934 $C_{22}H_{22}ClNNaO_2S$ requires M 422.0957.

General procedure for the batch addition of *N*-chloromorpholine (**16**) to *p*-anisaldehyde (**17**): To a round-bottomed flask with condenser, purged with nitrogen was added *N*-chloromorpholine (1 M in toluene). To this was then added the required equivalents of 4-anisaldehyde, *t* BuO_2H (70% aq.) and $FeCl_3{\cdot}6H_2O$. The reaction mixture was stirred under nitrogen at the required temperature and monitored by GC (calibrated to decane external standard). Then the reaction mixture was cooled to rt followed by the addition of saturated aqueous K_2SO_3. The product was extracted with Et_2O. The organic phases were combined, dried over Na_2SO_4, filtered and the solvent removed by rotary evaporation to give the crude as an orange oil. For a batch reaction with 77% conversion by ^1H NMR spectroscopy, isolation by chromatography gave the pure product in 54% yield. Reactions were monitored by calibrated GC analysis. GC method: H_2 10 psi, injector 250 °C, FID 250 °C, oven: 60 °C hold 3 min., 10 °C/min. to 200 °C, 200 °C hold 2 min. Amide: 16.7 min., aldehyde: 7.8 min, decane (external standard) 3.0 min. Sample preparation: 10 µL of 0.1 M decane in EtOAc, 10 µL reaction solution (organic phase), 980 µL MeOH. For GC calibration see below.

Continuous addition of *N*-chloromorpholine (**16**) to *p*-anisaldehyde (**17**): A round-bottomed flask connected to a reflux condenser and nitrogen supply. A solution of *N*-chloromorpholine (0.2 M) and 4-anisaldehyde (0.2 M where 1 equiv is required, 0.6 M where 3 equiv are required) in toluene was prepared and fed into the reactor by pump 1. A second solution of *t*-BuO_2H (70% aq.) was fed into the reaction by pump 2. Pump 3 was used to pump the reaction solution out of the reactor. The flow rates of the 3 pumps were chosen to give the required reactor volume, residence time and stoichiometry for the reaction, also, so that the flow rate of pump 3 (outflow from reactor) equalled the total flow rate of pumps 1 and 2 (reactor input). The reactor was heated to 110 °C and stirred under nitrogen. The reaction solution was collected in reactor volume (residence time) fractions. A small sample from each fraction was removed and analysed by GC to monitor conversion. Fractions collected at

steady state were combined and the product isolated as described for the general batch procedure above.

N-(4-Methoxybenzoyl)morpholine (**18**): Data compares with that previously reported for this compound [8]. ^{1}H NMR (500 MHz, $CDCl_3$) δ ppm 7.39 (2H, d J = 8.8 Hz, CHAr), 6.92 (2H, d J = 8.8 Hz, CHAr), 3.83 (3H, s, CH_3), 3.69-3.63 (8H, m, 4 × CH_2); ^{13}C NMR (125 MHz, $CDCl_3$) δ ppm 170.41 (CO), 160.91 (CAr), 129.21 (2 × CHAr), 127.36 (CAr), 113.80 (2 × CHAr), 66.94 (CH_3 + 2 × CH_2), 55.37 (2 × CH_2O).

General batch procedures for imine formation with Et_3N base: To a round-bottomed flask was added the required amount of *N*-chloramine in toluene (1 M). The solution was stirred at room temperature and to it was slowly added Et_3N (5 equiv). The reaction mixture was stirred for 42 hours. A saturated aqueous solution of $NaHCO_3$ was added to the mixture. The organic phase was separated, and the aqueous phase washed with EtOAc (3 washes). The organic phases were combined, dried over Na_2SO_4, filtered and the solvent removed by rotary evaporation to leave the crude product as a colourless oil, that was analysed by ^{1}HNMR.

General batch procedures for imine formation with NaOMe base: To a round-bottomed flask was added the required amount of *N*-chloramine in toluene (1 M). The solution was stirred at room temperature and to it was added an equal volume of MeOH followed by the slow addition of NaOMe (2 equiv). The reaction mixture was stirred, and conversion followed by ^{1}H NMR spectroscopy or GC analysis. Then MeOH was removed by rotary evaporation. To the residue was added water and EtOAc. The aqueous phase was removed and washed with further EtOAc (3 washes). The organic phases were combined, dried over Na_2SO_4, filtered and the solvent removed by rotary evaporation to leave the crude product as a colourless oil, which was analysed by ^{1}HNMR.

General batch procedures for imine formation with NaOH base: To a round-bottomed flask was added the required amount of *N*-chloramine in toluene (1 M). The solution was stirred at room temperature and to it was added an equal volume of aqueous NaOH (25% w/w) and tetrabutylammonium bromide (TBAB, 10 mol % relative to *N*-chloramine). The reaction mixture was stirred and conversion

followed by ^{1}H NMR spectroscopy or GC analysis. Once complete conversion of starting material was achieved, the organic phase was removed and dried by rotary evaporation to leave the crude product as a colourless oil. The reaction proceeded cleanly and further product purification was not required. The product was analysed by ^{1}HNMR.

General procedure for continuous imine formation with NaOMe base: The multi-stage CSTR was set up as outlined in Section 4.3 with the required number of stages and pumps. For homogenous solutions syringe pumps were used, for heterogeneous (slurry) solutions, a peristaltic pump was used. Two solutions were prepared: *N*-chloramine in toluene (1 M) and a slurry of NaOMe in MeOH (2 M). The solutions were fed into the reactor at equal flow rates such that the required residence time is achieved. The reaction solution was collected from the final stage of the reactor in reactor volume (1 residence time) fractions. A sample from each fraction was analysed by ^{1}H NMR spectroscopy or GC analysis to determine conversion.

General procedure for continuous imine formation with NaOH base: Three solutions were prepared: *N*-chloramine in toluene (1 M), aqueous NaOH (40% w/w) and TBAB in water (10 mol % TBAB relative to *N*-chloramine, volume of water = 0.5 × volume of *N*-chloramine solution). The solutions were fed into the reactor at flow rates such that the flow rate of each of the aqueous solutions is half that of the *N*-chloramine solution and the total flow rate gives the required residence time. For example, for a 10 mL reactor volume and a 10 min residence time the total flow rate should be 1 mL min^{-1} with the *N*-chloramine solution at 0.5 mL min^{-1}, and each of the aqueous solutions at 0.25 mL min^{-1}. The reaction solution was collected from the final stage of the reactor in reactor volume (1 residence time) fractions. A sample from each fraction was analysed by ^{1}H NMR spectroscopy or GC analysis to determine conversion. The TBAB and NaOH solutions should not be mixed via a T-piece and should be kept separate until entering the first CSTR stage. TBAB is not soluble in the NaOH solution and if these mix in a tube the TBAB precipitates and blocks the tube.

Presence of the amine as a byproduct of the reaction was confirmed with GC analysis. GC method: HP5 30 m, 0.32 mm, 0.25 µm. H_2 10 psi, injector 250 °C, FID 250 °C, oven: 60 °C hold 10 min,

5 °C/min to 100 °C, 100 °C hold 3 min, 50 °C/min to 300 °C. Imine 8.5 min., amine 4.6 min. Sample preparation: 10 µL reaction solution (organic phase), 990 µL MeOH.

Benzylidene(methyl)amine (**19**): Data compares with that previously reported for this compound [9]. ^{1}H NMR (500 MHz, $CDCl_3$) δ ppm 8.29 (1H, s, N=CH), 7.71-7.59 (2H, m, CHAr), 7.41-7.40 (3H, m, CHAr), 3.52 (3H, s, CH_3); ^{13}C NMR (125 MHz, $CDCl_3$) δ ppm 162.49 (CH=N), 136.27 (CAr), 130.51 (CHAr), 128.61 (2 × CHAr), 127.88 (2 × CHAr), 48.24 (CH_3). Calibrated GC analysis: HP5 30 m, 0.32 mm, 0.25 µm. H_2 10psi, injector 60 °C, FID 250 °C, oven: 40 °C hold 2 min, 5 °C/min to 70 °C, 70 °C hold 4 min, 100 °C/min to 250 °C, 250 °C hold 1 min. Imine 6 min., amine 6.9 min. dodecane (external standard) 10.7 min. Sample preparation: 10 µL of 0.1 M dodecane in EtOAc, 10 µL reaction solution (organic phase), 980 µL MeOH.

(*E*)-*N*-(α-Methylbenzylidene)methylamine (**20**): Data compares with that previously reported for this compound [10]. The ^{1}H NMR spectrum showed the imine to be present as a single isomer (*E*) with no evidence of a second set of peaks for the Z isomer. ^{1}H NMR (500 MHz, $CDCl_3$) δ ppm 7.76-7.74 (2H, m, CHAr), 7.38-7.36 (3H, m, CHAr), 3.35 (3H, s, CH_3N), 2.24 (3H, s, CH_3CN); ^{13}C NMR (125 MHz, $CDCl_3$) δ ppm 167.05 (C=N), 141.24 (CAr), 129.39 (CHAr), 128.21 (2 × CHAr), 126.43 (2 × CHAr), 39.52 (CH_3N), 15.10 (CH_3C).

2-Methyl-1,2-dehydropiperidine (**21**): Data compares with that previously reported for this compound [11]. ^{1}H NMR (500 MHz, $CDCl_3$) δ ppm 3.67-3.65 (m, 1H, CH_2NCl), 3.09-3.04 (m, 1H, CH^AH^B), 2.82 (br s, 1H, CH^AH^B), 2.82 (br s, 1H, CH), 1.84-1.61 (m, 4H, 2 × CH_2), 1.57-1.44 (m, 2H, CH_2), 1.38 (d J = 6.1 Hz, CH_3); ^{13}C NMR (125 MHz, $CDCl_3$) δ ppm 168.11 (C=N), 49.25 (CH_2), 30.27 (CH_2), 27.60 (CH_2), 21.68 (CH_2), 21.48 (CH_2), 19.65 (CH_2).

Iridium-catalyzed asymmetric reduction of (*E*)-*N*-(α-methylbenzylidene)methylamine (**20**): To a nitrogen-purged flask was added (*E*)-*N*-(α-methylbenzylidene)methylamine (135 mg, 1 mmol), $[IrCp^*Cl_2]_2$ (4 mg, 0.005 mmol), (*R*,*R*)-TsDPEN (4 mg, 0.01 mmol). To this mixture was then added formic acid/triethylamine 5:2 (1 mL). The reaction mixture was stirred under nitrogen at 40 °C for

2 hours. Following this the reaction solution was filtered through silica (1:1 EtOAc/petroleum ether 40–60) and the resulting solution dried by rotary evaporation to leave the crude product as a pale yellow oil. The crude was analysed by chiral GC to confirm formation of the product and to determine conversion and enantiomeric excess.

GC method: CP-Chirasil-Dex-CB, 25 m 0.25 mm, 0.25 μm. H_2 7.5 psi, injector 250 °C, FID 250 °C, oven: 80 °C hold 1 min, 100 °C/min to 100 °C, 13 min hold, 100 °C/min to 200 °C, 5 min hold. (*R*)-amine 16.6 min. (*S*)-amine 17.1 min.

S4.2 Single-stage heated CSTR assembly

A three-necked round-bottomed flask equipped with a condenser was employed as a laboratory scale CSTR and was heated using an aluminium heating block. Reactants were pumped into the CSTR through one neck and out of the opposing neck at equal flow rate to maintain a constant volume, via $^{1}/_{16}$" OD PTFE tubing (see Figure S3).

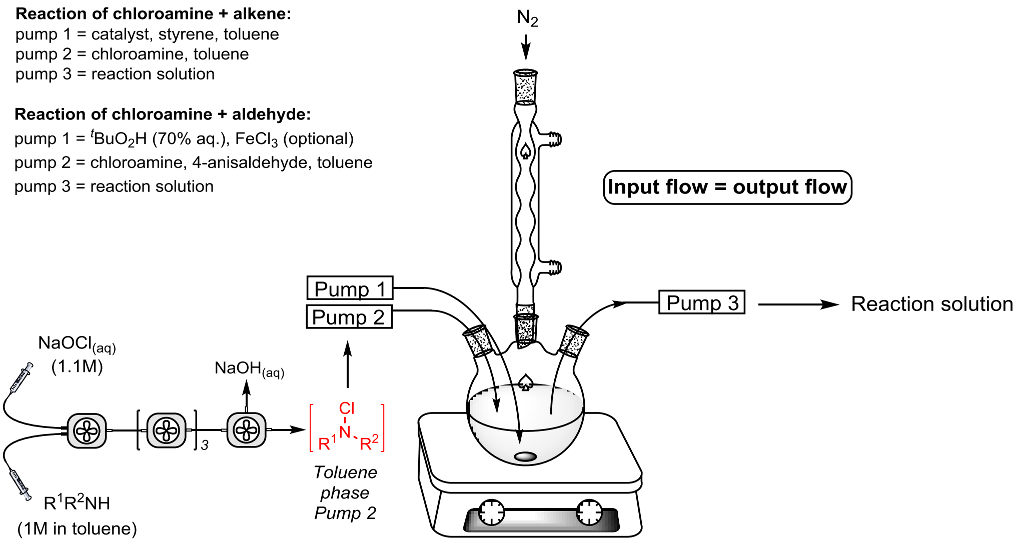

Figure S3. Single-stage heated CSTR configuration.

S4.3 Multi-stage CSTR assembly

A laboratory scale cascade CSTR developed within our group (and described elsewhere) [4] was employed to generate and dispense *N*-chloroamines as described within the manuscript. For clarity, the reactor assembly is reproduced in Figure S4 below.

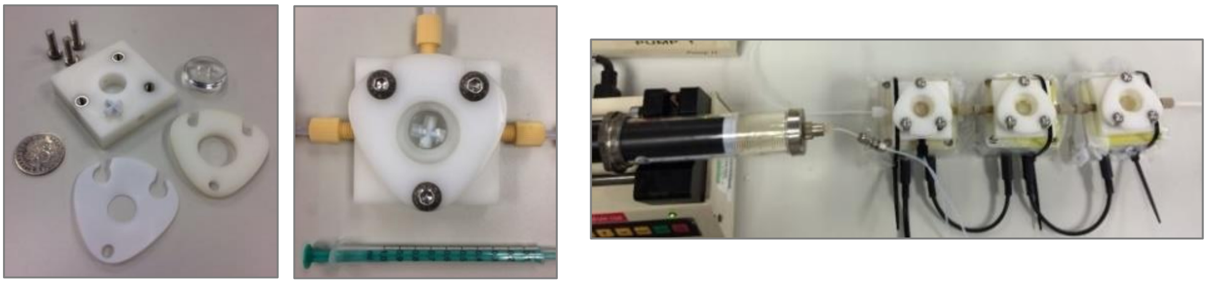

Figure S4. Left: individual components of reactor (coin for scale). Middle: assembled single CSTR unit. Right: 3-stage reactor unit.

S4.4 NaOCl titration

Acetic acid (1 mL) and potassium iodide (350 mg) were diluted to 50 mL using DI water. To this solution was added NaOCl solution as obtained from Sigma-Aldrich (1 mL). $Na_2S_2O_3$ (0.1 M) was then titrated into the aqueous solution of potassium iodide, acetic acid and NaOCl until the orange solution became colourless. The concentration of the initial NaOCl solution was determined using the equation supplied below:

$$HOCl + 2I^- \rightarrow I_2 + Cl^- + OH^-$$

$$I_2 + 2S_2O_3^{2-} \rightarrow S_4O_6^{2-} + 2I^-$$

S5. GC conditions and calibration profiles

GC Method: HP-5 column 30 m × 0.32 mm × 0.25 μm, H2 10 psi, split ratio 5:1, T = 35 °C 3 min, 10 °C/min to 200 °C, 200 °C 3 min, 280 °C 3 min.

Morpholine: 2.2 min

Piperidine: 1.8 min

N-Benzylmethylamine: 7.1 min

N-Methyl-p-toluenesulfonamide: 6.1 min

Dibutylamine: 3.4 min

Dibenzylamine: 16.2 min

Toluene: 1.5 min.

Calibrations

p-Anisaldehyde:

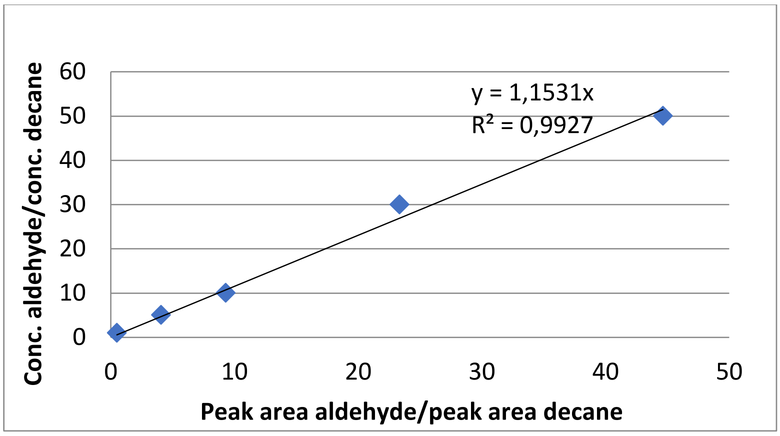

N-(4-Methoxybenzoyl)morpholine:

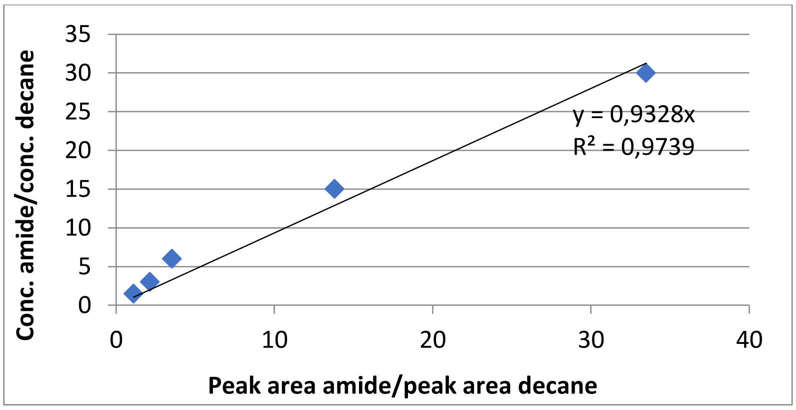

N-Benzylmethylamine:

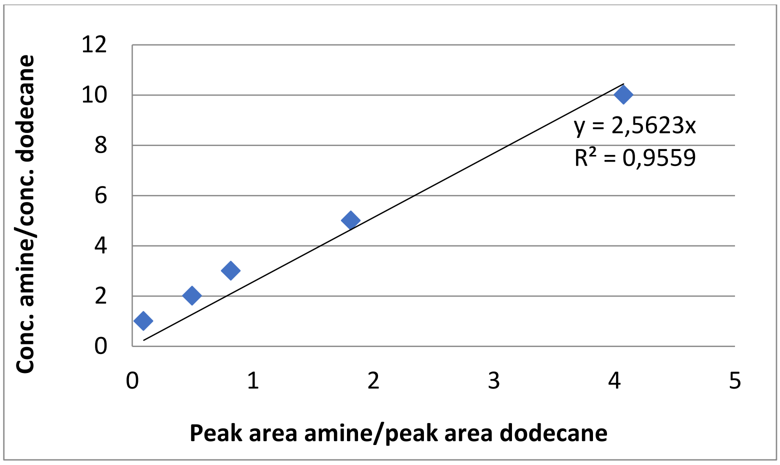

N-Benzylidenemethylamine:

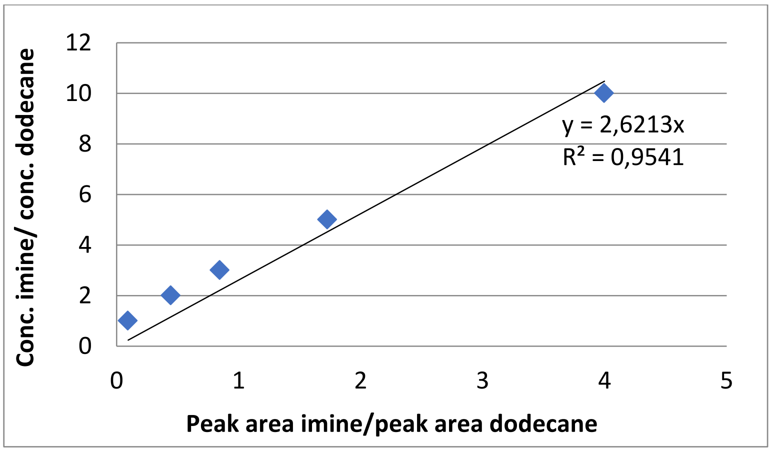

S6. Relevant ^{1}H and ^{13}C NMR spectra.

All ^{1}H and ^{13}C NMR spectra are supplied below and were recorded using $CDCl_3$ reference solvent.

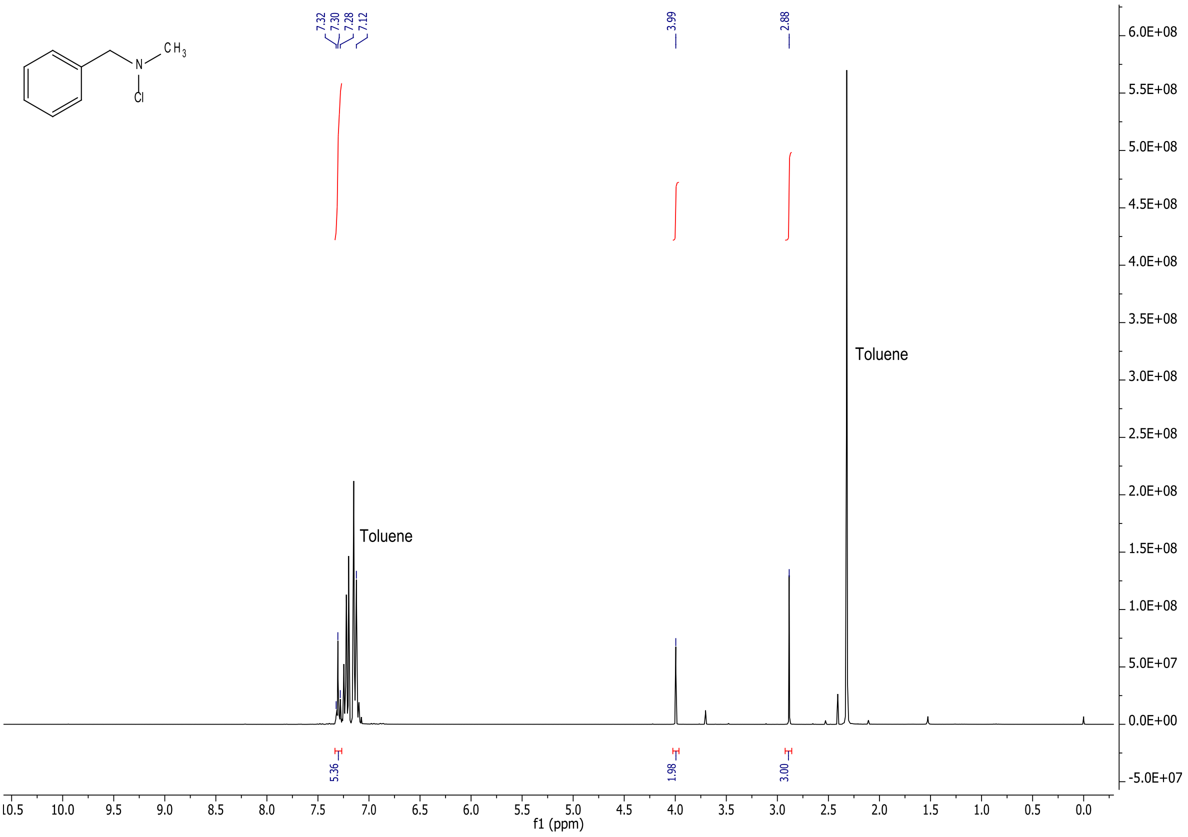

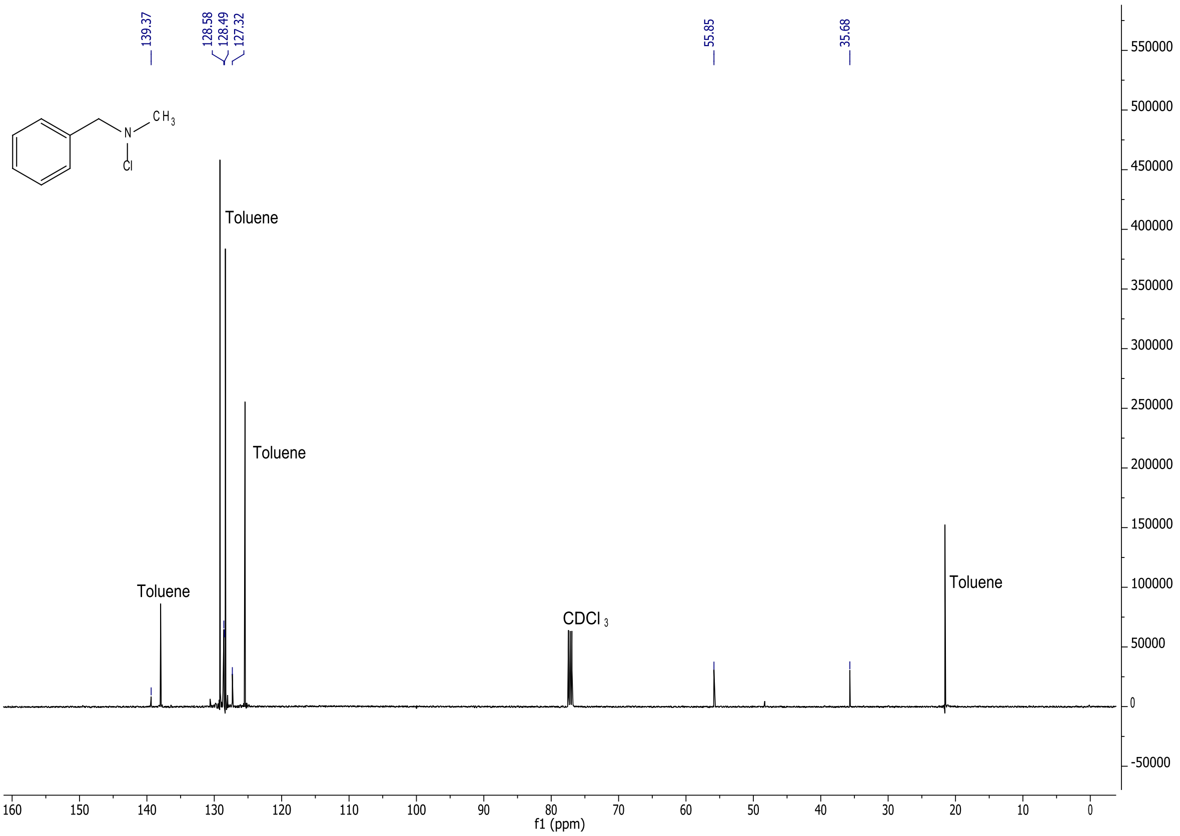
139.37
128.58
128.49
127.32
55.85
35.68
CH3
N
Cl
Toluene
Toluene
Toluene
CDCl3
Toluene
f1 (ppm)

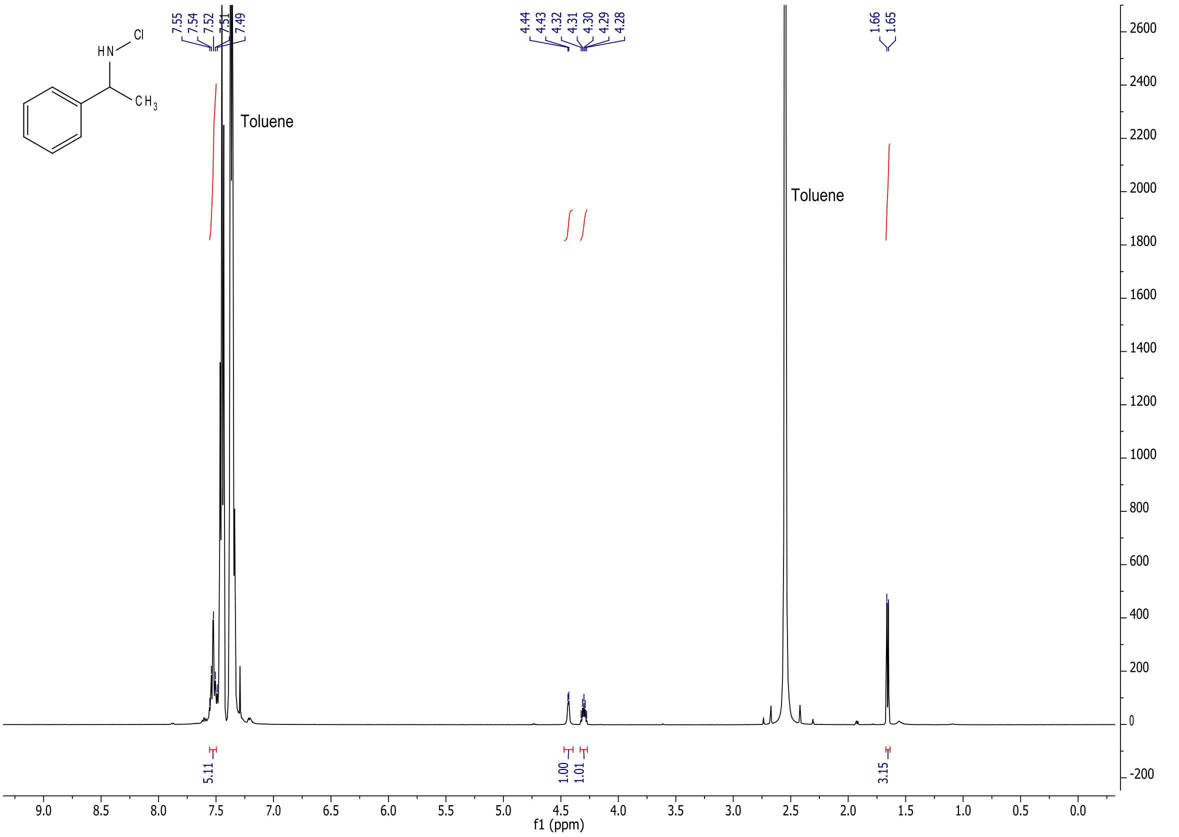
7.55
7.54
7.52
7.51
7.49
4.44
4.43
4.32
4.31
4.30
4.29
4.28
1.66
1.65
HN
Cl
CH3
Toluene
Toluene
5.11
1.00
1.01
3.15
f1 (ppm)

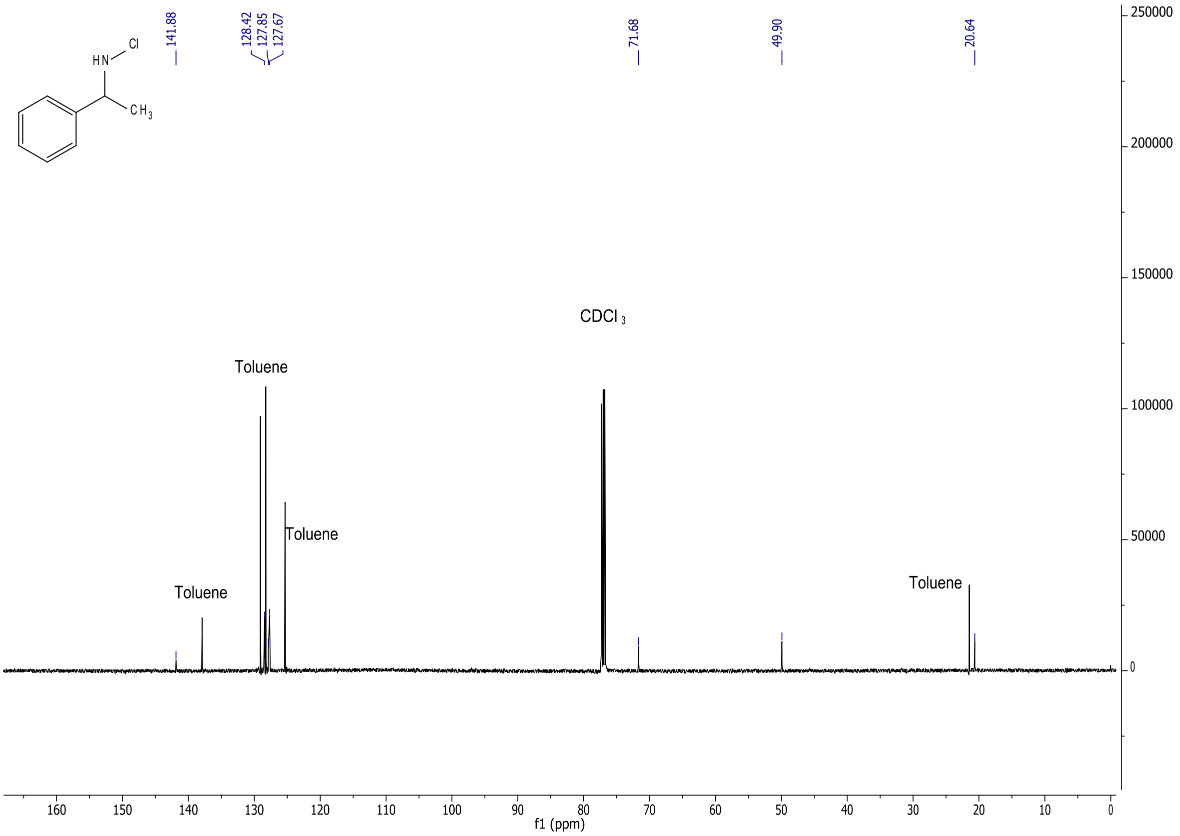

141.88
128.42
127.85
127.67
71.68
49.90
20.64
CDCl 3
Toluene
Toluene
Toluene
Toluene
f1 (ppm)

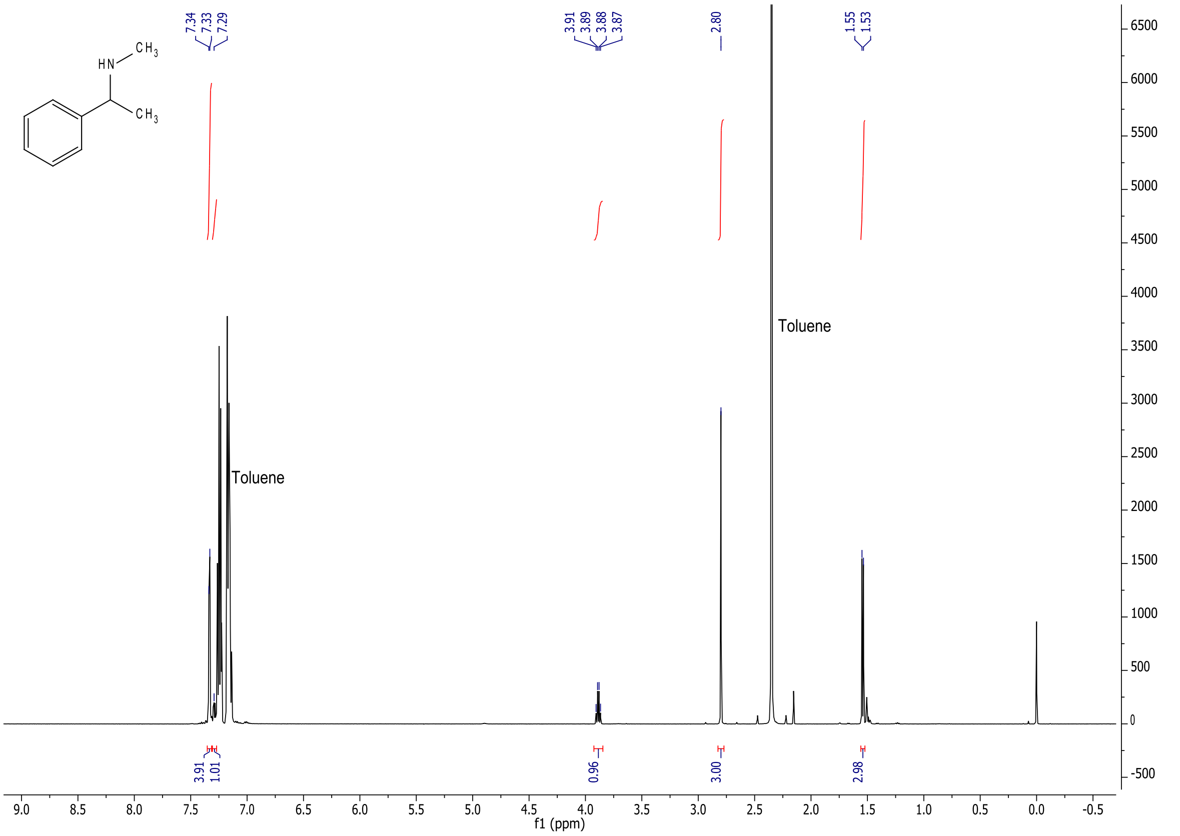

7.34
7.33
7.29
3.91
3.89
3.88
3.87
2.80
1.55
1.53
Toluene
Toluene
3.91
1.01
0.96
3.00
2.98
f1 (ppm)

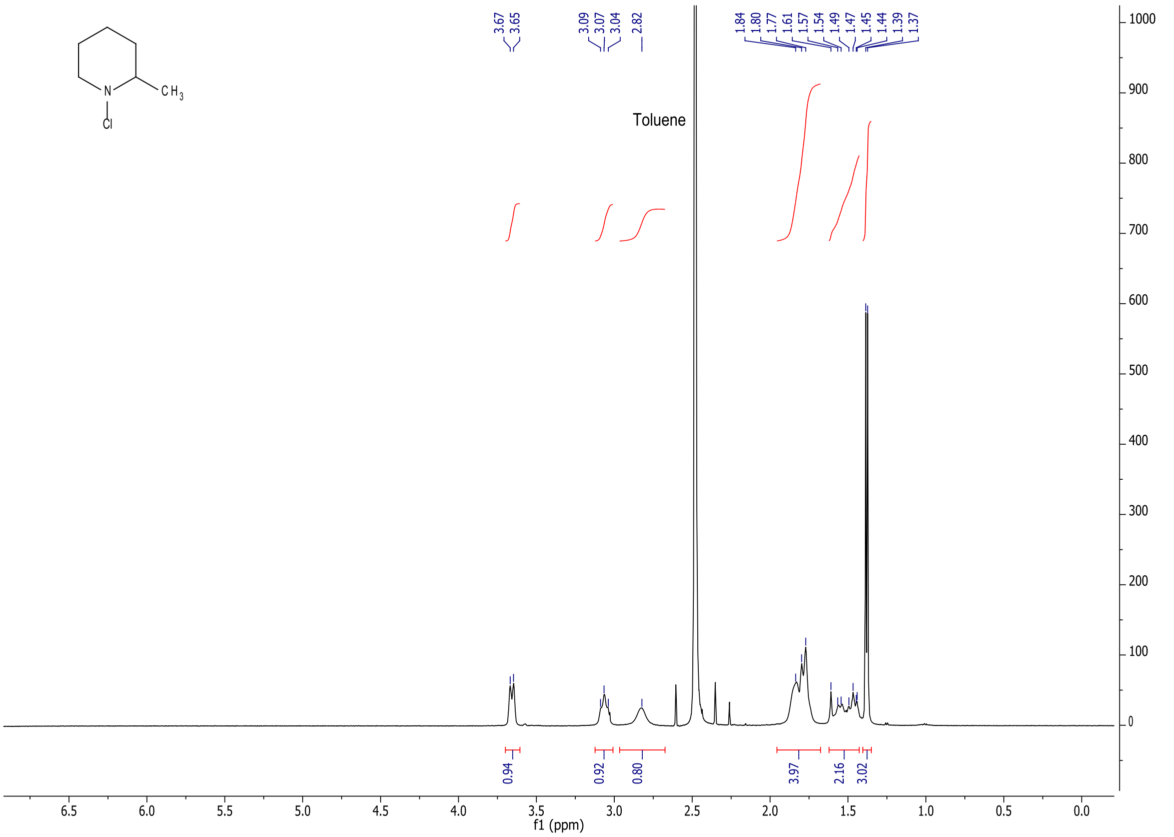
Cl
N
CH3
Toluene
3.67
3.65
3.09
3.07
3.04
2.82
1.84
1.80
1.77
1.61
1.57
1.54
1.49
1.47
1.45
1.44
1.39
1.37
0.94
0.92
0.80
3.97
2.16
3.02
f1 (ppm)

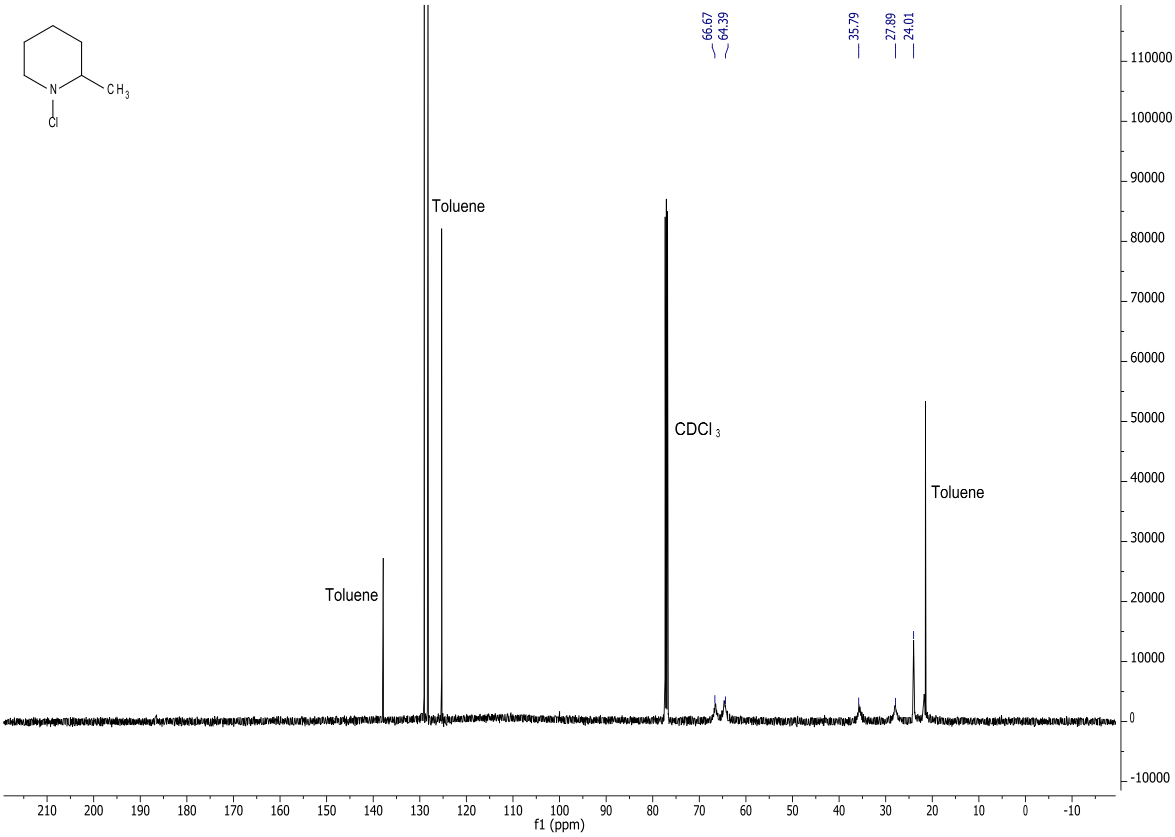
Cl
N
CH3
66.67
64.39
35.79
27.89
24.01
Toluene
CDCl 3
Toluene
Toluene
f1 (ppm)

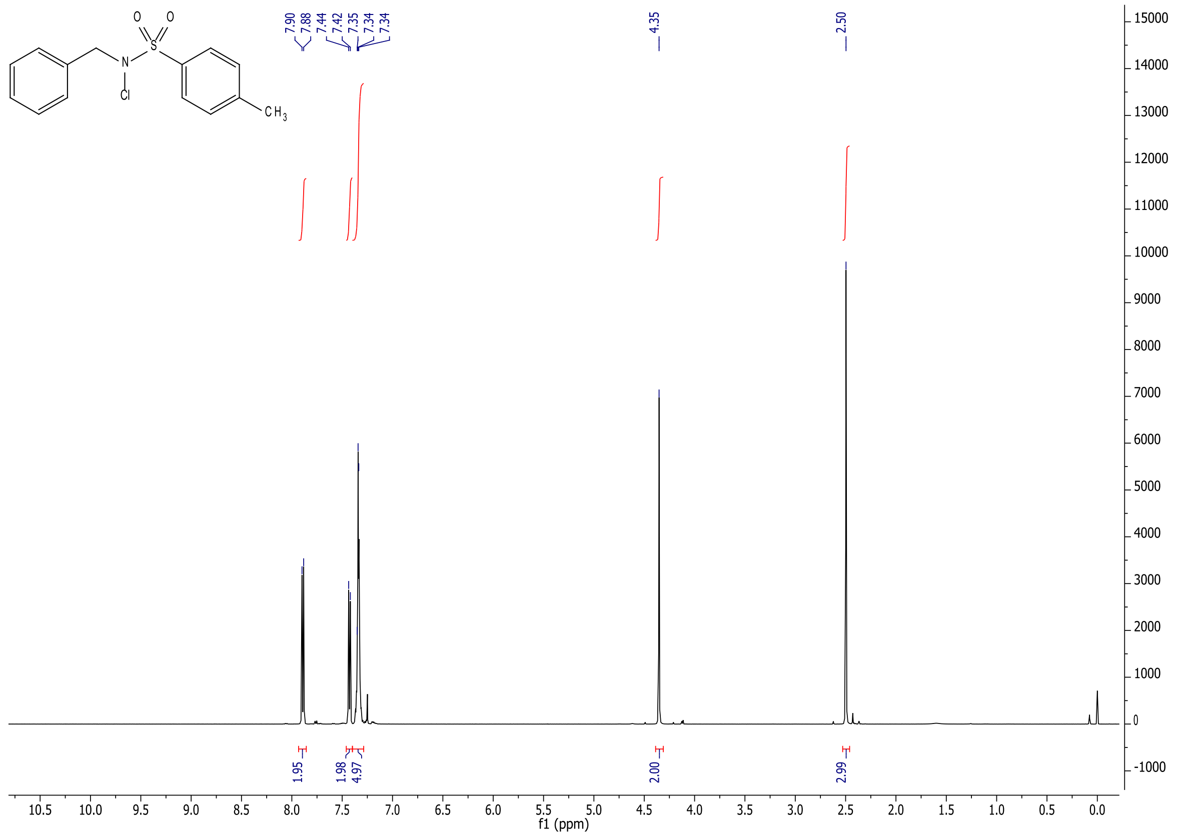
7.90
7.88
7.44
7.42
7.35
7.34
7.34
4.35
2.50
1.95
1.98
4.97
2.00
2.99
f1 (ppm)

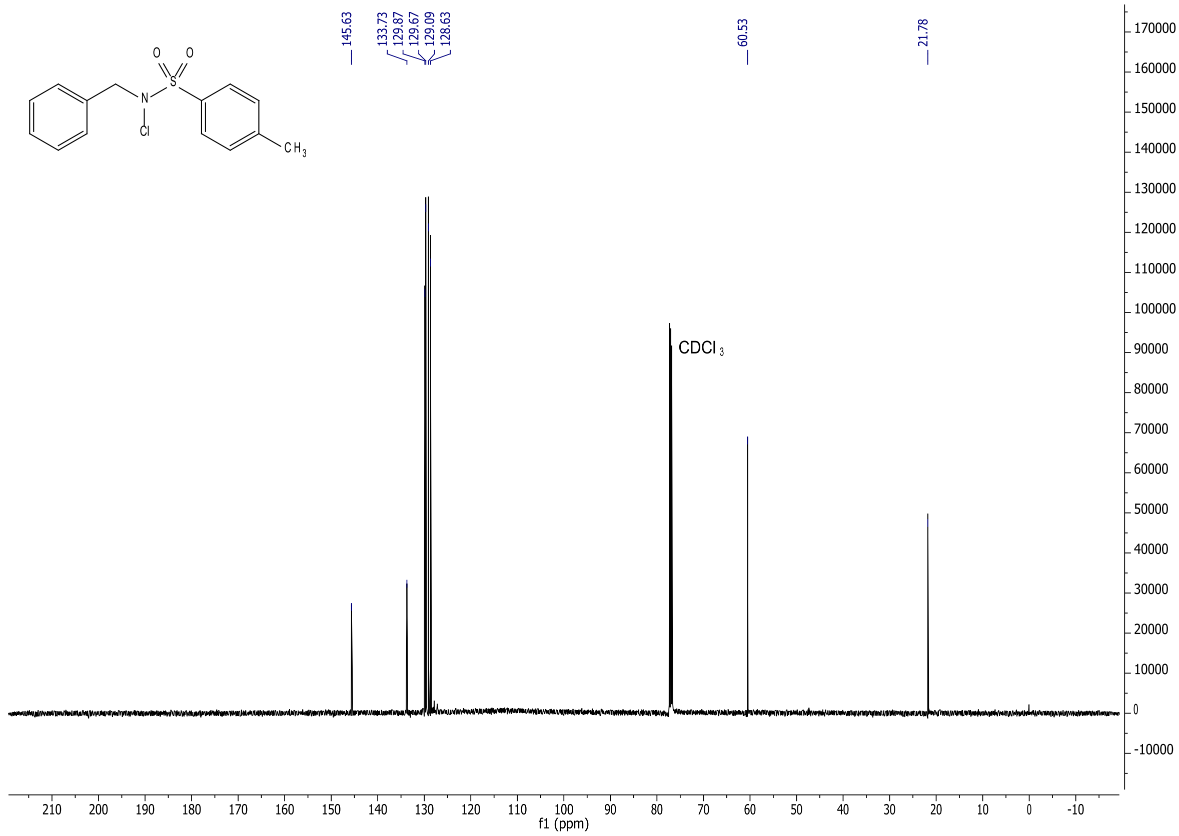
145.63
133.73
129.87
129.67
129.09
128.63
60.53
21.78
CDCl3
f1 (ppm)

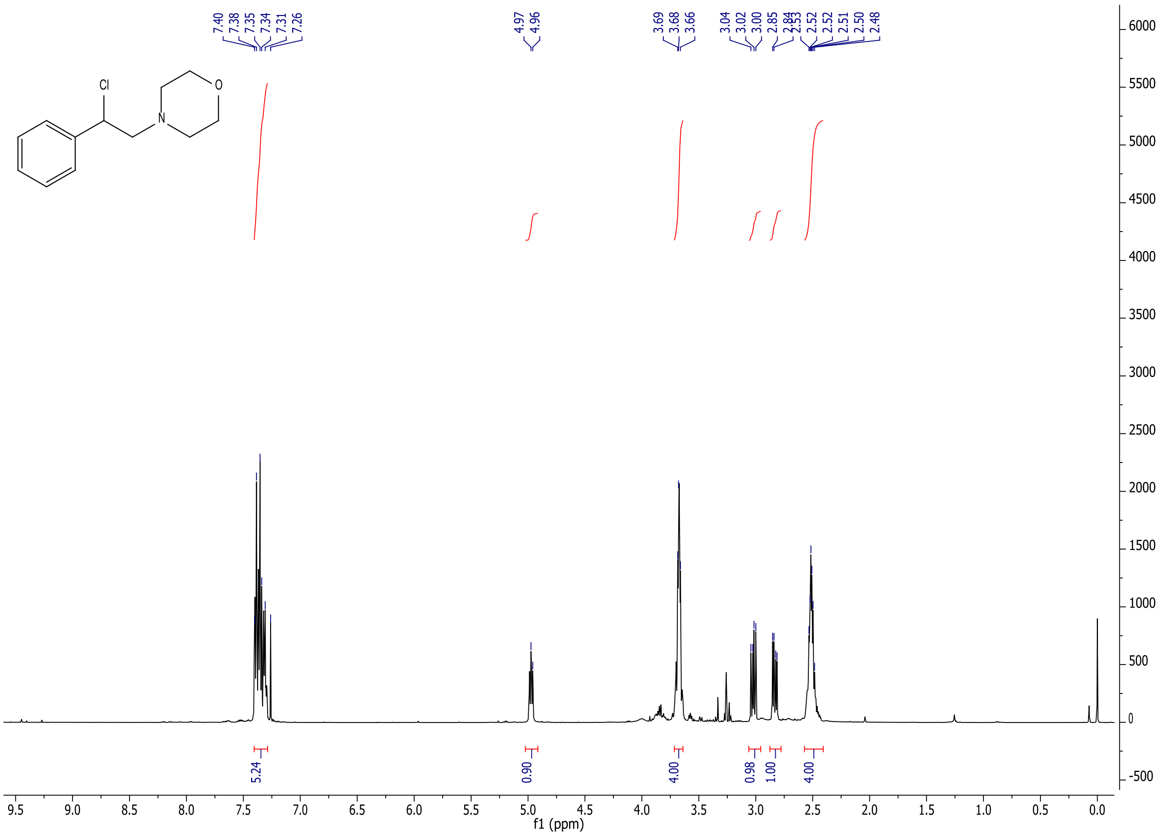
7.40 7.38 7.35 7.34 7.31 7.26
4.97 4.96
3.69 3.68 3.66
3.04 3.02 3.00 2.85 2.84 2.53 2.52 2.52 2.51 2.50 2.48
Cl
N
O
5.24
0.90
4.00
0.98
1.00
4.00
f1 (ppm)

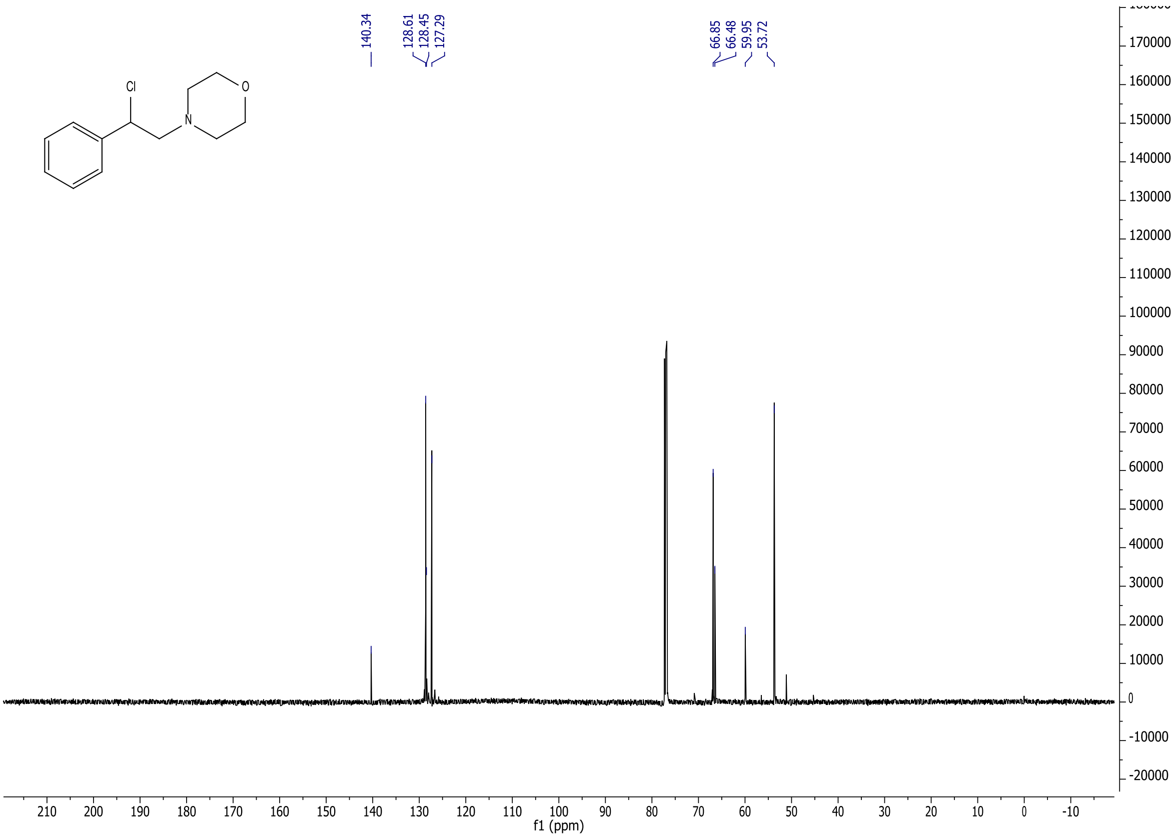
140.34
128.61 128.45 127.29
66.85 66.48 59.95 53.72
Cl
N
O
f1 (ppm)

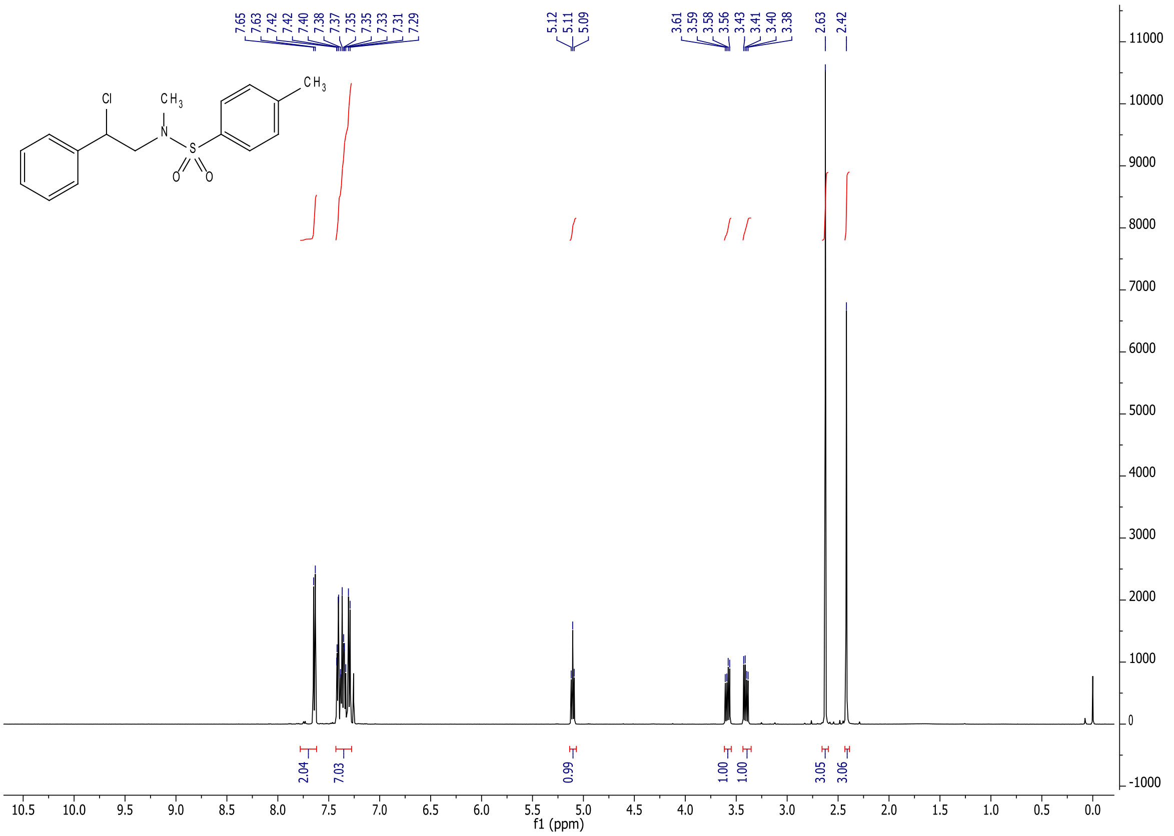
7.65
7.63
7.42
7.42
7.40
7.38
7.37
7.35
7.35
7.33
7.31
7.29
5.12
5.11
5.09
3.61
3.59
3.58
3.56
3.43
3.41
3.40
3.38
2.63
2.42
Cl
CH3
N
S
O
O
CH3
2.04
7.03
0.99
1.00
1.00
3.05
3.06
f1 (ppm)

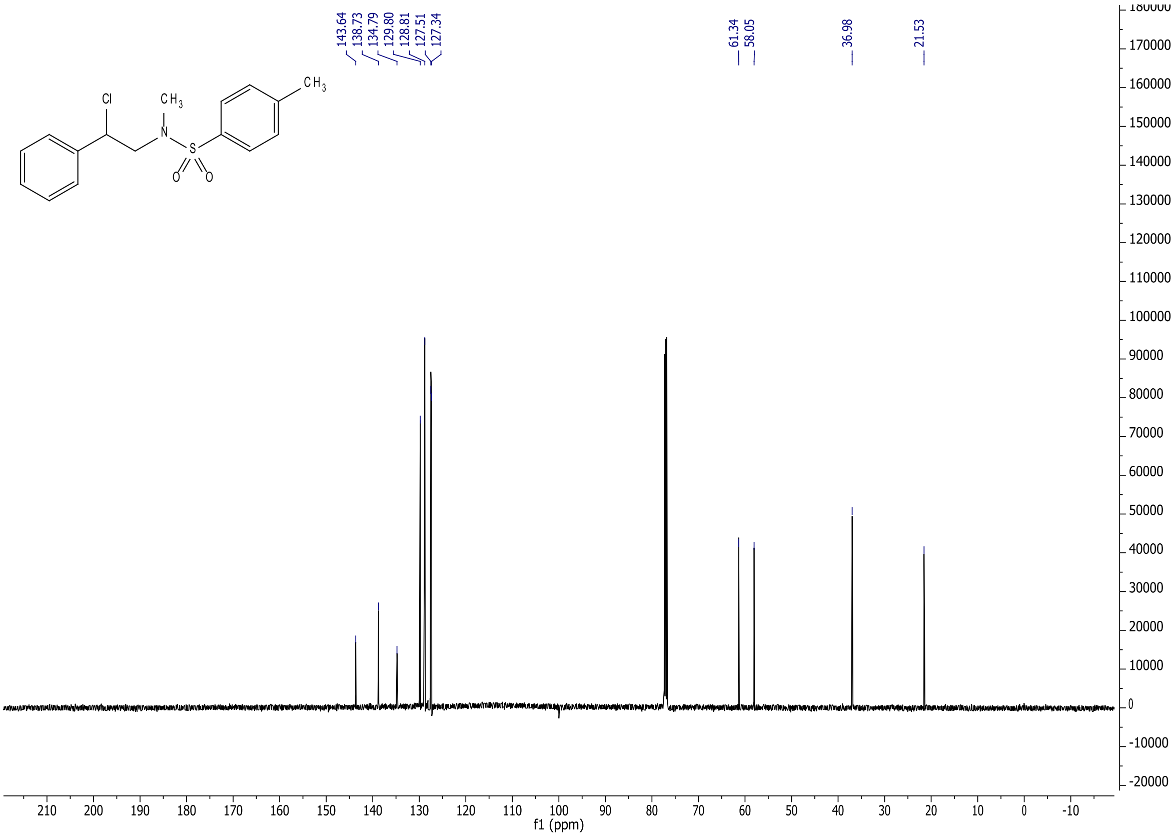
143.64
138.73
134.79
129.80
128.81
127.51
127.34
61.34
58.05
36.98
21.53
Cl
CH3
N
S
O
O
CH3
f1 (ppm)

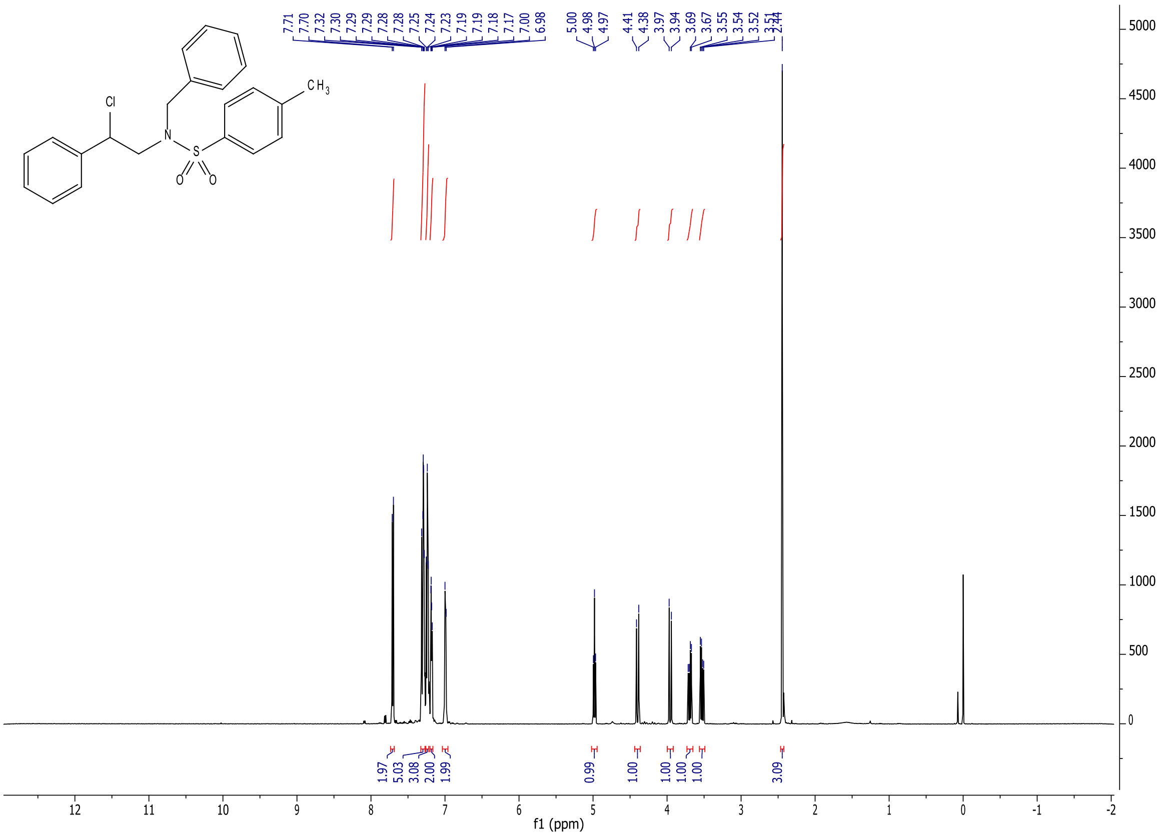
7.71
7.70
7.32
7.30
7.29
7.29
7.28
7.28
7.25
7.24
7.23
7.19
7.19
7.18
7.17
7.00
6.98
5.00
4.98
4.97
4.41
4.38
3.97
3.94
3.69
3.67
3.55
3.54
3.52
3.51
2.44
Cl
N
S
O
O
CH3
1.97
5.03
3.08
2.00
1.99
0.99
1.00
1.00
1.00
1.00
3.09
f1 (ppm)

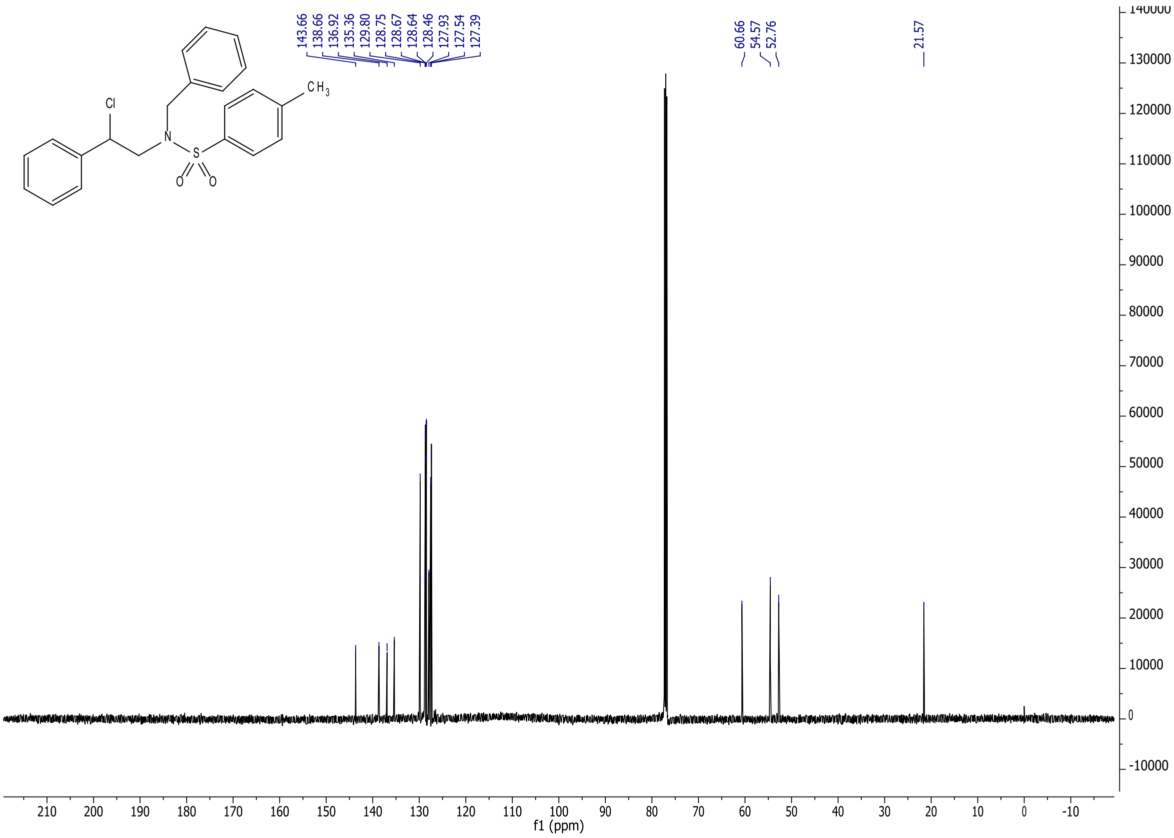
143.66
138.66
136.92
135.36
129.80
128.75
128.67
128.64
128.46
127.93
127.54
127.39
60.66
54.57
52.76
21.57
Cl
N
S
O
O
CH3
f1 (ppm)

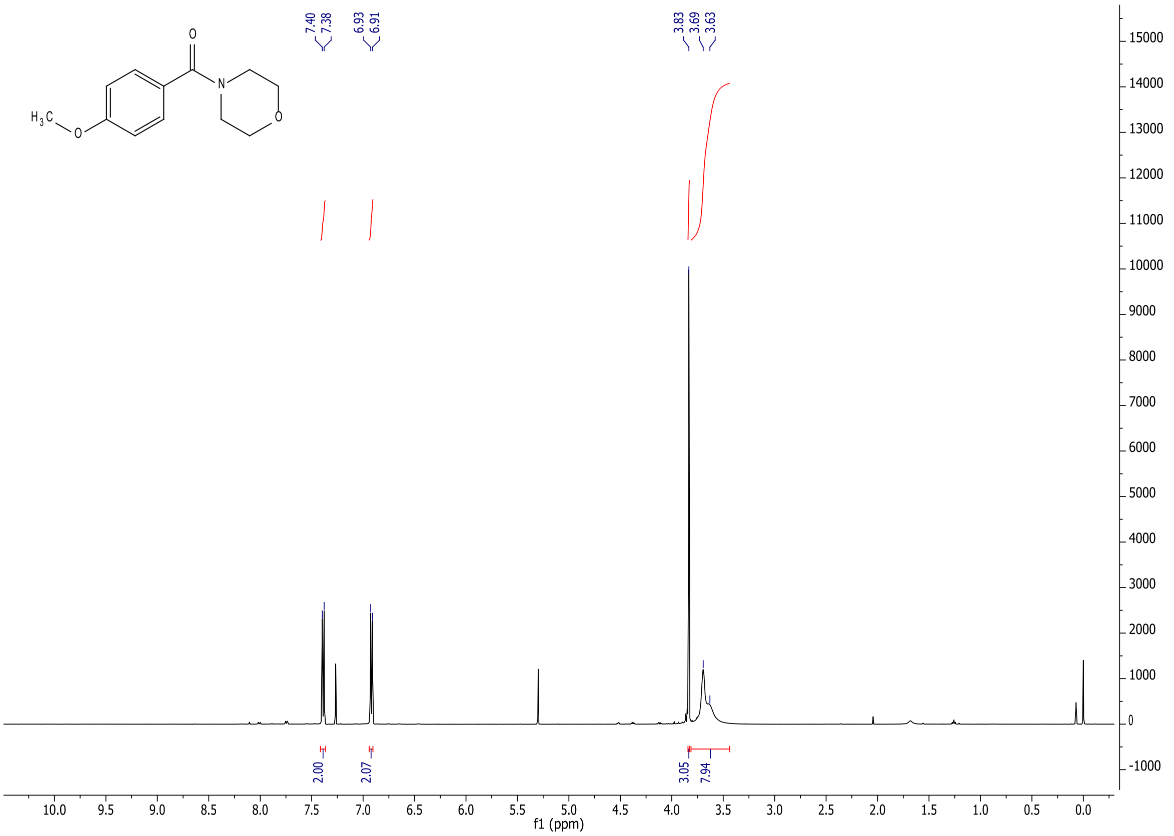
7.40
7.38
6.93
6.91
3.83
3.69
3.63
H3C
O
N
2.00
2.07
3.05
7.94
f1 (ppm)

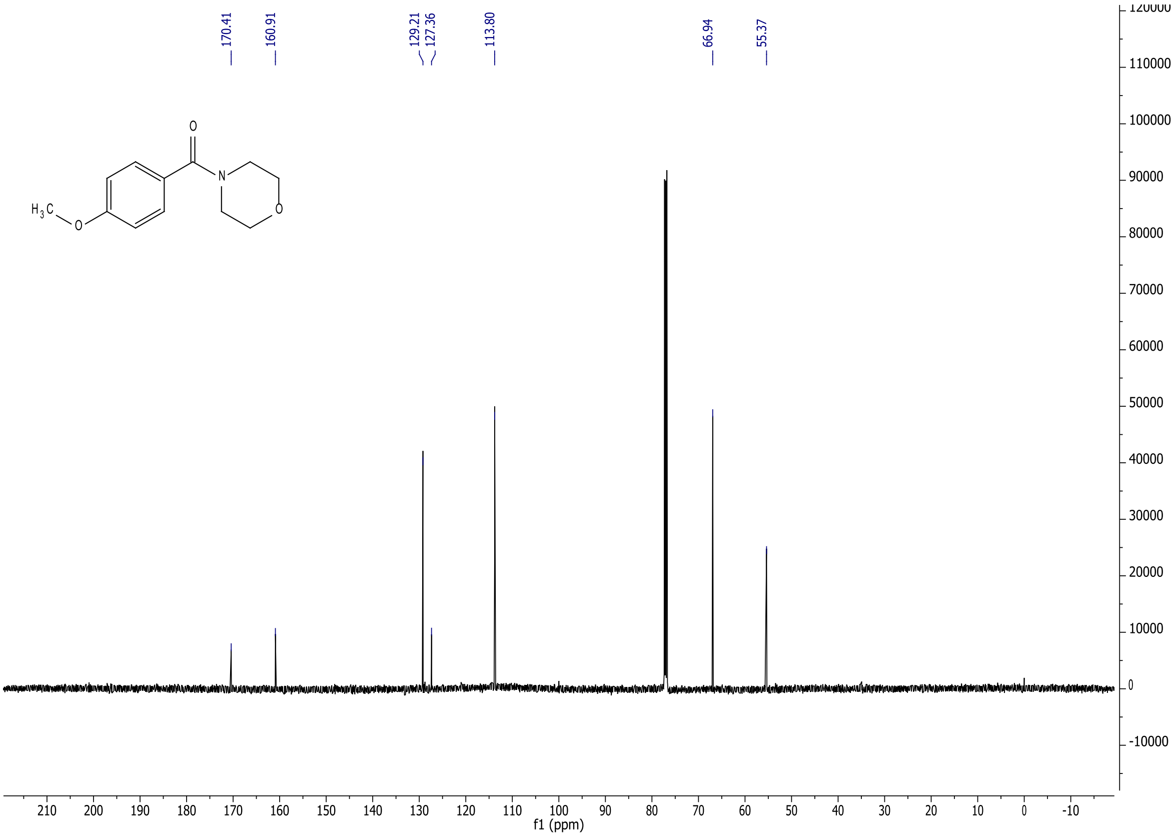
170.41
160.91
129.21
127.36
113.80
66.94
55.37
H3C
O
N
f1 (ppm)

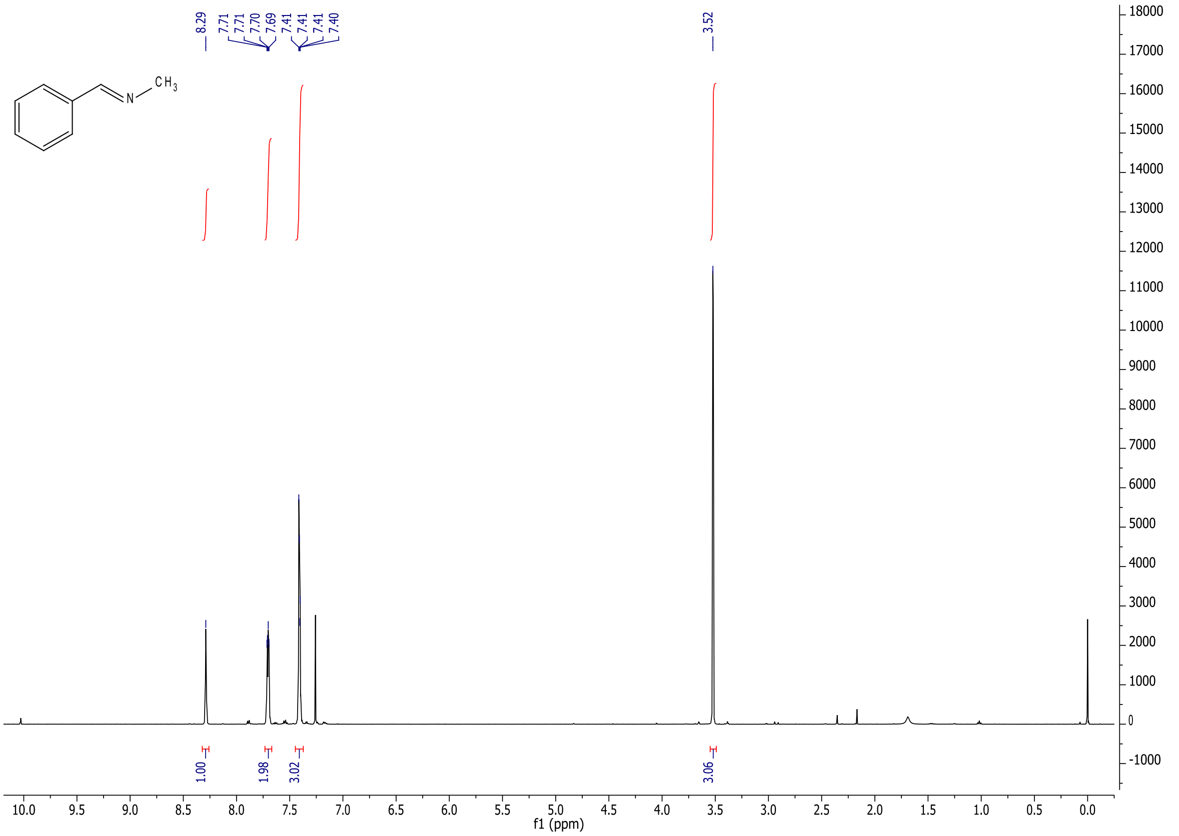

8.29
7.71
7.71
7.70
7.69
7.41
7.41
7.41
7.40
3.52
CH3
N
1.00
1.98
3.02
3.06
f1 (ppm)

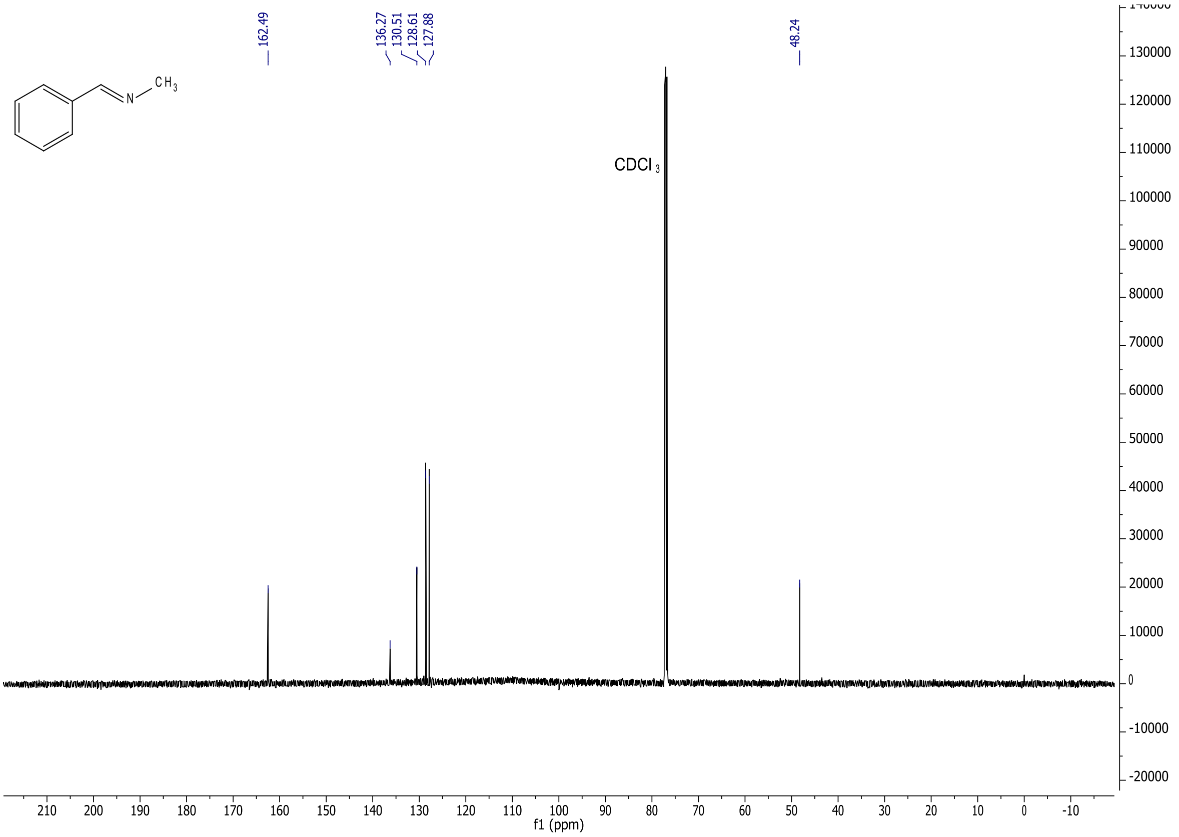

162.49
136.27
130.51
128.61
127.88
48.24
CH3
N
CDCl3
f1 (ppm)

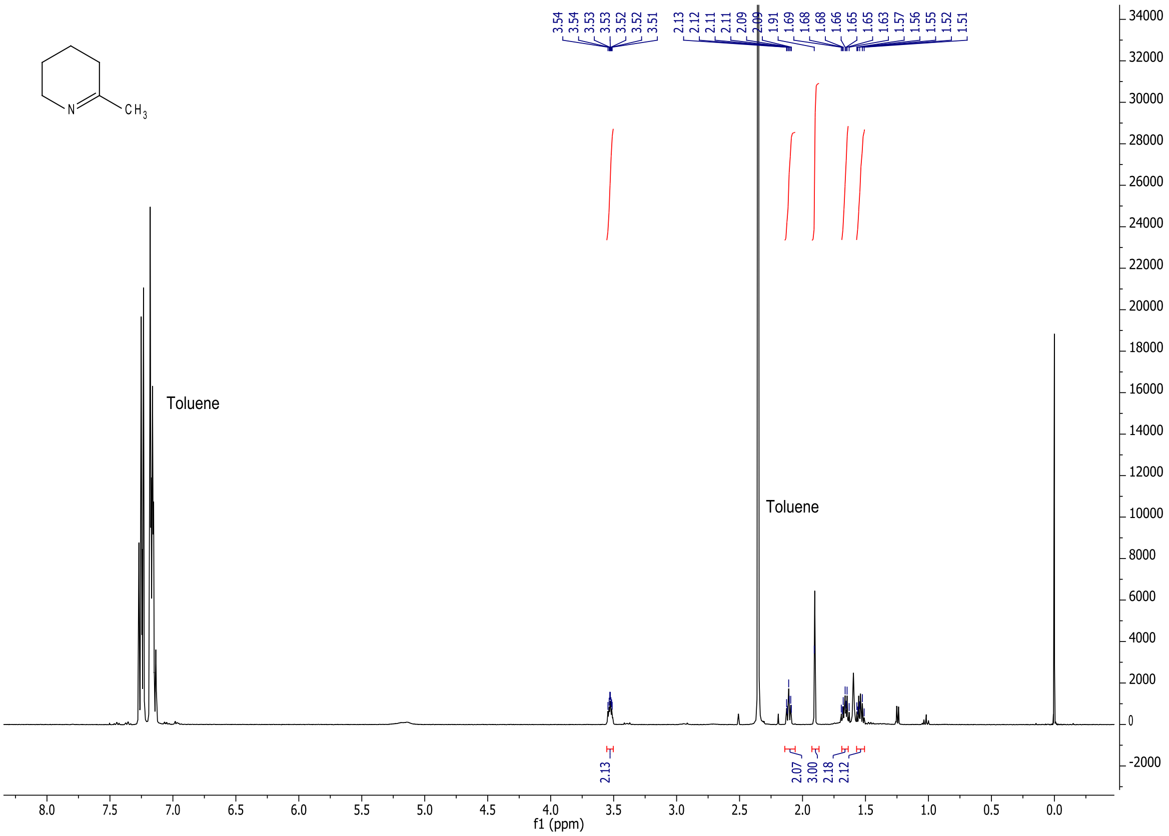
3.54
3.54
3.53
3.53
3.52
3.52
3.51
2.13
2.12
2.11
2.11
2.09
2.09
1.91
1.69
1.68
1.68
1.66
1.65
1.65
1.63
1.57
1.56
1.55
1.52
1.51
N
CH3
Toluene
Toluene
2.13
2.07
3.00
2.18
2.12
f1 (ppm)

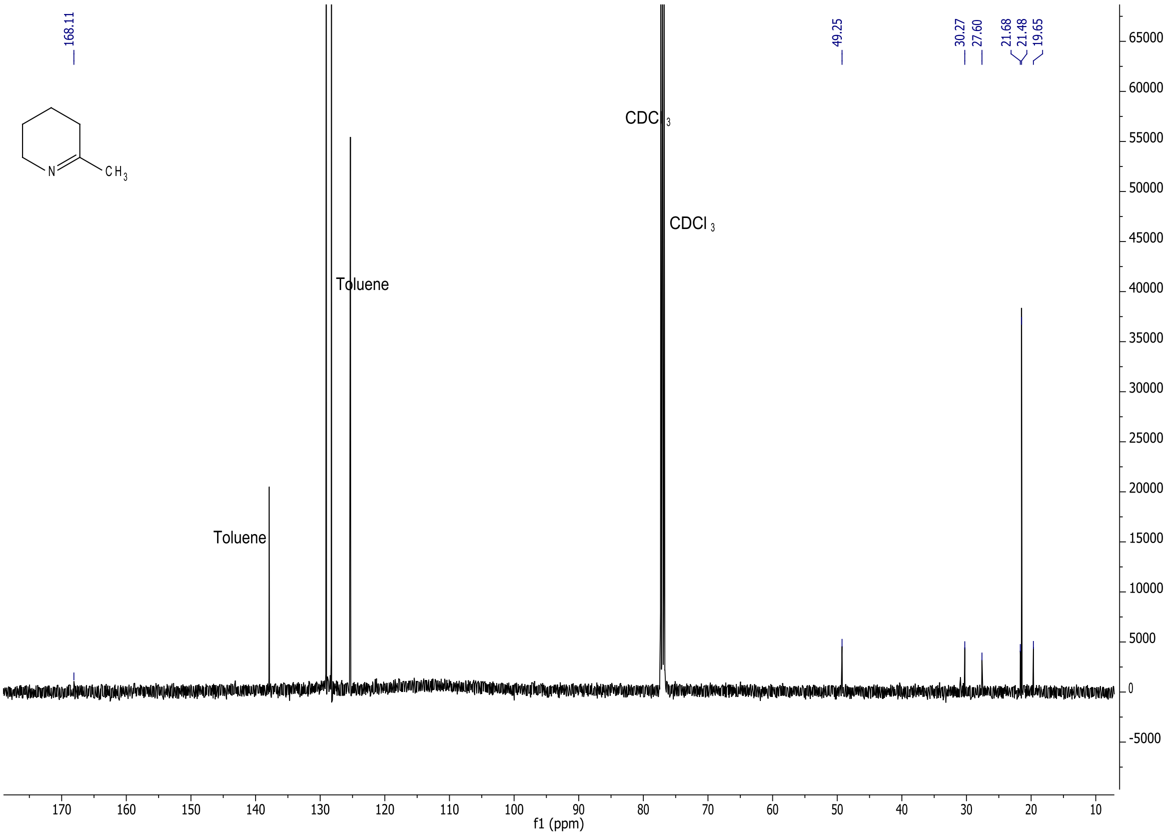
168.11
49.25
30.27
27.60
21.68
21.48
19.65
N
CH3
CDCl3
CDCl3
Toluene
Toluene
f1 (ppm)

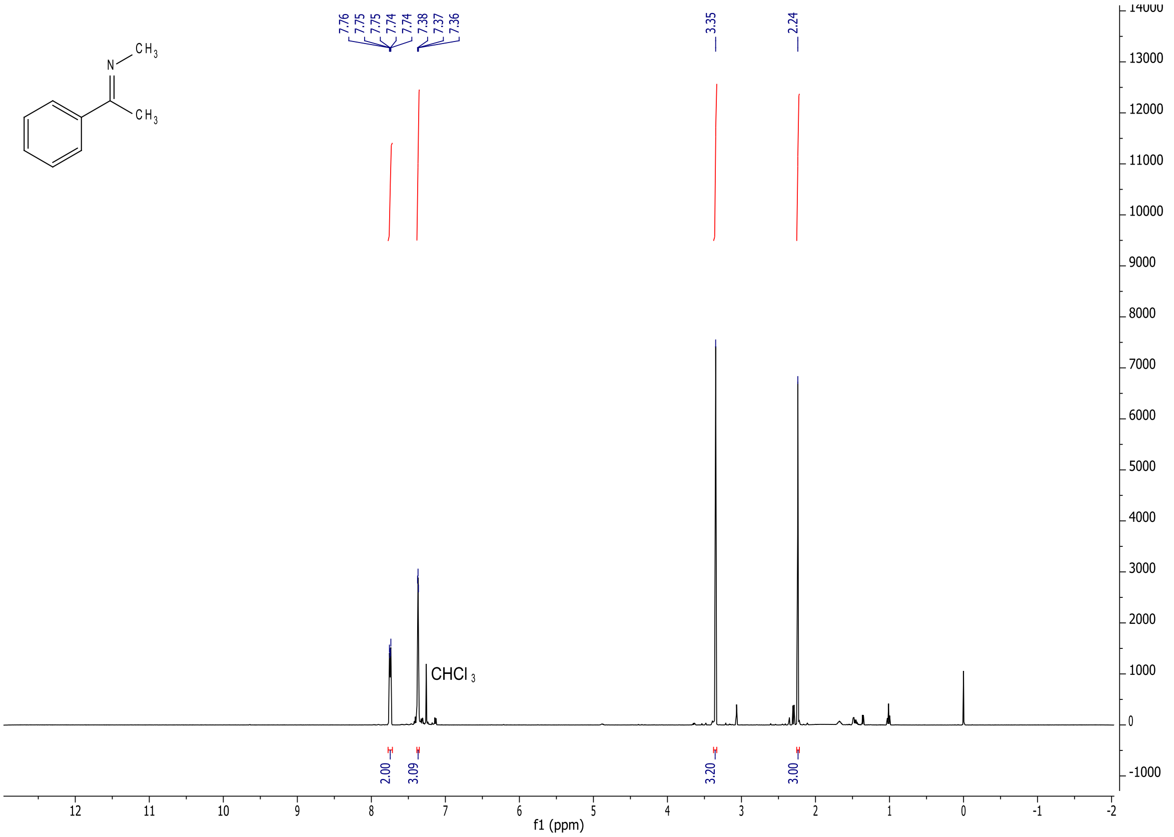
7.76 7.75 7.75 7.74 7.74 7.38 7.37 7.36
3.35
2.24
N
CH3
CH3
CHCl 3
2.00
3.09
3.20
3.00
f1 (ppm)

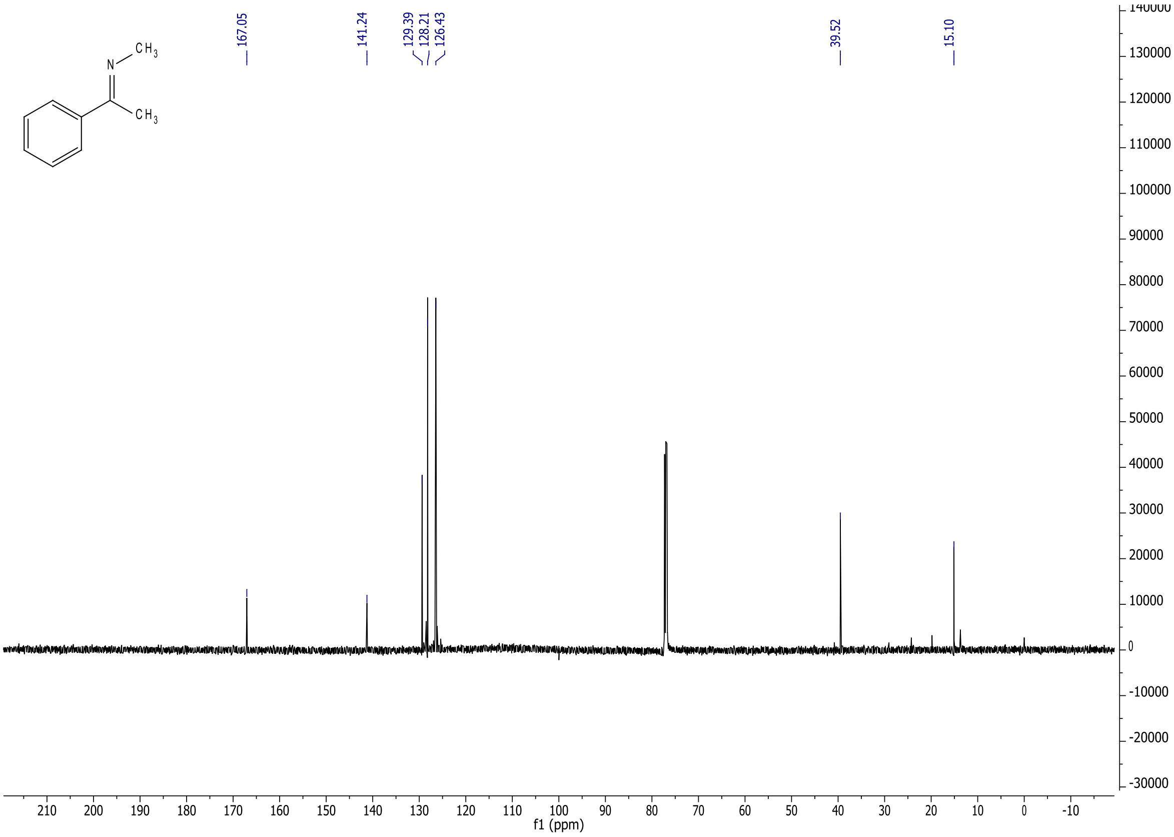
167.05
141.24
129.39 128.21 126.43
39.52
15.10
N
CH3
CH3
f1 (ppm)

7. References

[1] M. T. Bovino, S. R. Chemler, Angew. Chem. Int. Ed. 2012, 51, 3923-3927.

[2] Q. Li, M. Shi, C. Timmons, G. Li, Org. Lett. 2006, 8, 625-628.

[3] Blacker, A. J.; Jolley, K. E. *Beilstein J. Org. Chem.* **2015**, *11*, 2408-2417.

[4] M. R. Chapman, M. H. Kwan, G. King, K. E. Jolley, M. Hussain, S. Hussain, I. E. Salama, C. González Nino, L. A. Thompson, M. E. Bayana, A. Clayton, B. N. Nguyen, N. J. Turner, N. Kapur, A. J. Blacker, Org. Process Res. Dev. 2017, 21, 1294–1301.

[5] Barker, T. J.; Jarvo, E. R. *J. Am. Chem. Soc.* **2009**, *131*, 15598-15599.

[6] Zhong, Y. -L.; Zhou, H.; Gauthier, D. R.; Lee, J.; Askin, D.; Dolling, U. H.; Volante, R. P. *Tetrahedron Lett.* **2005**, *46*, 1099-1101.

[7] Davis, B. G.; Maughan, M. A. T.; Chapman, T. M.; Villard, R.; Courtney, S. *Org. Lett.* **2002**, *4*, 103-106.

[8] Porcheddu, A.; de Luca, L. *Adv. Synth. Catal.* **2012**, *354*, 2949-2953.

[9] Knöpke, L. R.; Nemati, N.; Köckritz, A.; Brückner, A.; Bentrup, U. *ChemCatChem* **2010**, *2*, 273-280.

[10] Samec, J. S. M.; Mony, L.; Bäckvall, J.-E. *Can. J. Chem.* **2005**, *83*, 909-916

[11] Van, T. N.; De Kimpe, N. *Tetrahedron* **2000**, *56*, 7969-7973.
